# Common genetic variation in *ETV6* is associated with colorectal cancer susceptibility

**DOI:** 10.1038/ncomms11478

**Published:** 2016-05-05

**Authors:** Meilin Wang, Dongying Gu, Mulong Du, Zhi Xu, Suzhan Zhang, Lingjun Zhu, Jiachun Lu, Rui Zhang, Jinliang Xing, Xiaoping Miao, Haiyan Chu, Zhibin Hu, Lei Yang, Cuiju Tang, Lei Pan, Haina Du, Jian Zhao, Jiangbo Du, Na Tong, Jielin Sun, Hongbing Shen, Jianfeng Xu, Zhengdong Zhang, Jinfei Chen

**Affiliations:** 1 Department of Oncology, Nanjing First Hospital, Nanjing Medical University, Nanjing 210006, China; 2 Jiangsu Key Laboratory of Cancer Biomarkers, Prevention and Treatment, Collaborative Innovation Center for Cancer Personalized Medicine, Nanjing Medical University, Nanjing 211166, China; 3 Department of Genetic Toxicology, Key Laboratory of Modern Toxicology of Ministry of Education, School of Public Health, Nanjing Medical University, Nanjing 211166, China; 4 State Key Laboratory of Reproductive Medicine, Nanjing Medical University, Nanjing 210029, China; 5 Department of Surgical Oncology, Second Affiliated Hospital, Zhejiang University School of Medicine, Hangzhou 310009, China; 6 Department of Oncology, First Affiliated Hospital of Nanjing Medical University, Nanjing 210029, China; 7 Institute for Chemical Carcinogenesis, State Key Lab of Respiratory Disease, Guangzhou Medical University, Guangzhou 510182, China; 8 Department of Colorectal Surgery, Liaoning Cancer Hospital and Institute, Shenyang 110042, China; 9 Department of Cell Biology and Cell Engineering Research Center, State Key Laboratory of Cancer Biology, Xijing Hospital, Fourth Military Medical University, Xi'an 710032, China; 10 Department of Epidemiology and Biostatistics, School of Public Health, Tongji Medical College, Huazhong University of Science and Technology, Wuhan 430030, China; 11 Department of Epidemiology and Biostatistics, School of Public Health, Nanjing Medical University, Nanjing 211166, China; 12 Program for Personalized Cancer Care, NorthShore University Health System, Evanston, Illinois 60201, USA

## Abstract

Genome-wide association studies (GWASs) have identified multiple susceptibility loci for colorectal cancer, but much of heritability remains unexplained. To identify additional susceptibility loci for colorectal cancer, here we perform a GWAS in 1,023 cases and 1,306 controls and replicate the findings in seven independent samples from China, comprising 5,317 cases and 6,887 controls. We find a variant at 12p13.2 associated with colorectal cancer risk (rs2238126 in *ETV6, P*=2.67 × 10^−10^). We replicate this association in an additional 1,046 cases and 1,076 controls of European ancestry (*P*=0.034). The G allele of rs2238126 confers earlier age at onset of colorectal cancer (*P*=1.98 × 10^−6^) and reduces the binding affinity of transcriptional enhancer MAX. The mRNA level of *ETV6* is significantly lower in colorectal tumours than in paired normal tissues. Our findings highlight the potential importance of genetic variation in *ETV6* conferring susceptibility to colorectal cancer.

## Abstract (web summary)

Genome-wide association studies have been performed to identify genetic variants that are associated with susceptibility to colorectal cancer. Here, the authors expand on these studies and identify a variant that regulates the expression of ETV6 and find that over-expression of ETV6 blocks cell proliferation *in vitro*.

## Graphical abstract

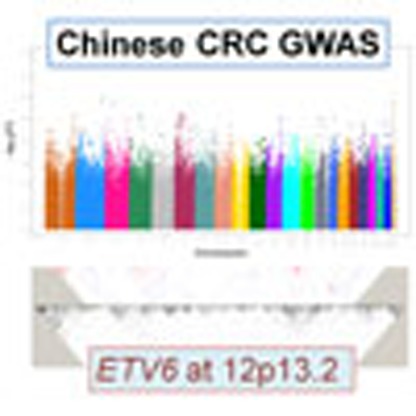

Colorectal cancer is the third most common cancer and the fourth leading cause of cancer-related mortality, comprising more than 1.2 million new cases and 0.6 million deaths each year^1^. Colorectal cancer is a common complex disease caused by environmental and genetic factors and their interactions. Twin and family studies have shown that inherited genetic factors play an essential role in the predisposition to colorectal cancer and are responsible for ∼35% of the colorectal cancer risk^2^. However, less than 5% of total colorectal cancer cases are explained by particular high penetrance genes, such as the DNA mismatch repair genes, *APC*, *SMAD4* and *MUTYH*^3^. Therefore, the remaining unidentified heritability may be attributable to common variants with low penetrance.

Genome-wide association studies (GWASs) in populations of European ancestry have revealed over 20 susceptibility loci associated with colorectal cancer risk^4^,^5^,^6^,^7^,^8^,^9^,^10^,^11^,^12^,^13^. However, many of these variants show only weak or no effects among Asians, suggesting the presence of genetic heterogeneity between European and Asian ethnicities^14^,^15^. Recently, a GWAS of colorectal cancer in East Asians identified 11 novel loci for colorectal cancer risk, indicating a genetic basis for colorectal cancer in East Asians as well^16^,^17^,^18^,^19^. However, these loci identified thus far account for only ∼7.7% of the genetic risk of colorectal cancer among East Asians^19^. Therefore, to search for additional susceptibility regions for colorectal cancer in Asians, we undertook a multistage GWAS across eight independent cohorts that included 14,533 Han Chinese subjects (Fig. 1). Here we report 12p13.2 as a new susceptibility locus for colorectal cancer and provide new insights into the genetic aetiology of colorectal cancer.

## Results

### New susceptibility locus for colorectal cancer

The characteristics of the included subjects in each study are summarized in Supplementary Table 1. After standard quality control for single-nucleotide polymorphisms (SNPs) and individuals, 691,326 SNPs in 1,023 cases and 1,306 controls were selected for further association analysis. Principal-component analysis (PCA) revealed that all study subjects were Han Chinese, with modest evidence of population stratification in the study populations (Supplementary Fig. 1). A quantile–quantile plot revealed that the inflation factor (*λ*) was 1.067 (Supplementary Fig. 2).

A Manhattan plot for the association between each SNP and colorectal cancer risk is shown in Supplementary Fig. 3. Across the genome, multiple loci showed suggestive evidence for association, although no SNP exceeded the genome-wide significance threshold with a *P*<5 × 10^−8^. The association between known SNPs based on previously reported colorectal cancer GWAS and colorectal cancer risk was evaluated for all samples (Supplementary Table 2). Three variants (rs6691170, rs16892766 and rs3217810) were not polymorphic among Asians. Among the other SNPs, 11 were significantly associated with colorectal cancer in the same direction as described previously (*P*_additive_<0.05). The reported risk alleles of all those 11 SNPs were associated with increased risk for colorectal cancer, with odds ratios (ORs) ranging between 1.14 and 1.44.

To examine the suggestive associations obtained from the GWAS stage, we selected 53 SNPs for the replication stage based on the following criteria: (i) the SNPs had *P*_additive_<1 × 10^−3^ in the GWAS stage; (ii) only one SNP with the lowest association was selected among multiple SNPs strong linkage disequilibrium (LD) of *r*^2^>0.5; (iii) the SNPs displaying strong LD (*r*^2^>0.5) with previously reported associated loci were excluded. The associations between the 53 selected SNPs and colorectal cancer risk are shown in Supplementary Table 3.

Except for one SNP, rs929271, all of the selected SNPs were successfully genotyped in an additional case–control study comprising 855 cases and 1,258 controls (Nanjing-2, China; Supplementary Table 4). Of the 52 SNPs analysed, three SNPs (rs418410, rs3122160, and rs2238126) were nominally significantly associated with colorectal cancer risk at *P*<0.05. However, only rs2238126 at 12p13.2 showed a significant association consistent in direction with the GWAS stage (*P*_additive_=4.46 × 10^−3^). To confirm the significance of this association, we genotyped rs2238126 in additional Han Chinese populations, including 4,462 cases and 5,629 controls from six independent study centres (Wuhan, Guangzhou, Nanjing-3, Xi'an, Hangzhou and Shenyang). We conducted a combined analysis of the initial GWAS and replication studies and found that the rs2238126 G allele had an increased risk of colorectal cancer (*P*_additive_=2.67 × 10^−10^, OR=1.17; Table 1). There was no significant heterogeneity among the eight study groups (*P*_het_=0.626, *I*^2^=0; Supplementary Fig. 4).

**Figure 2:**
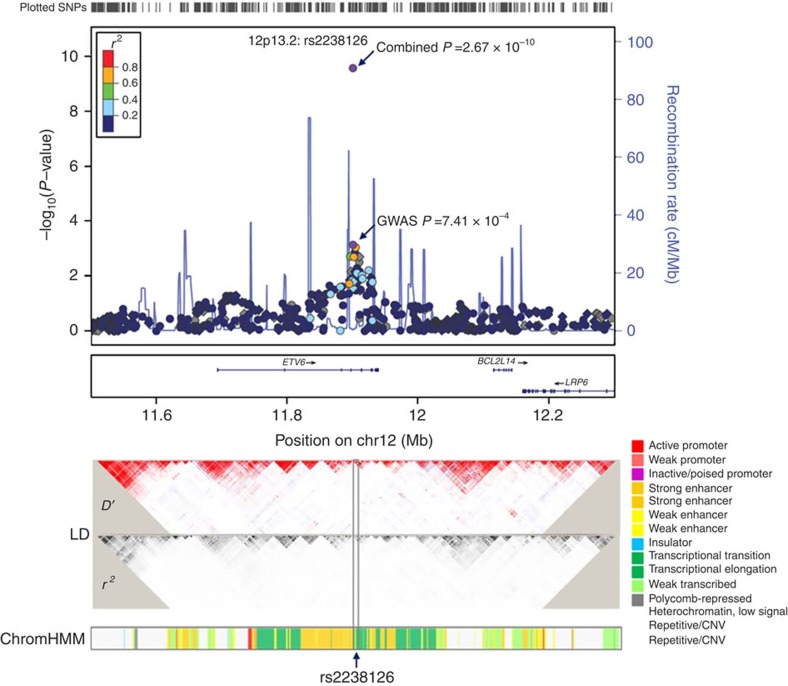
Region association plot of rs2238126 at 12p13.2 for colorectal cancer. In the top panel of the region plot, the association results (−log_10_ *P*) of both genotyped (circle) and imputed (diamond) SNPs in the GWAS samples are shown for SNPs in the region 400 kb upstream and downstream of rs2238126. Imputation was performed on this region using the 1,000 Genomes Project CHB and JPT data as a reference. The genes within the region of interest are indicated by arrows. The right *y* axis represents the recombination rate between the SNPs. The LD plots (*D*' and *r*^2^) estimated based on the CHB and JPT populations are shown in the middle panel. The bottom panel represents the chromatin state segmentation track (ChromHMM) from GM12878 lymphoblastoid cells.

To further characterize colorectal cancer-associated SNPs at 12p13.2, we performed an imputation from the 1,000 Genomes Project as a reference (Fig. 2). We measured the associations between imputed SNPs (imputed *r*^2^>0.1, minor allele frequency (MAF)>0.05 and within 400 kb on either side of rs2238126) and colorectal cancer risk and identified 37 additional SNPs that were significant at *P*_additive_<0.05 (Supplementary Table 5). However, rs2238126 showed the strongest association, and no residual association with other SNPs was detected when controlling for the effect of rs2238126 in this region.

We further investigated the effect of rs2238126 on colorectal cancer risk by a subgroup analysis (Supplementary Table 6). As shown in Supplementary Fig. 5, we did not observe significant differences between subgroups in age (*P*_het_=0.729), sex (*P*_het_=0.318), smoking status (*P*_het_=0.537) or tumour site (*P*_het_=0.567). However, analysis of the age at diagnosis among colorectal cancer cases revealed that individuals with the GG genotype had a 2.2-year earlier age at diagnosis than those with the AA genotype (Supplementary Fig. 6). Regression analysis revealed that the rs2238126 G allele was significantly associated with earlier age at onset of colorectal cancer (effect=−1.007 year per allele, combined *P*=1.98 × 10^−6^; Supplementary Table 7).

### Association analysis in European colorectal cancer GWAS

We also evaluated the association between rs2238126 and colorectal cancer risk in an European population of 1,046 cases and 1,076 controls from the Ontario Familial Colorectal Cancer Registry. As shown in Table 2, the rs2238126 G allele showed a significant risk effect in the same direction among Europeans (OR=1.19, *P*_additive_=0.034). The combined analysis of European and Asian populations showed a stronger association, with a *P* value of 2.79 × 10^−11^. Nevertheless, the G allele frequency of rs2238126 in the European population differed considerably from that in the Chinese population.

### Potential regulatory role of rs2238126 on *ETV6*

**Figure 3:**
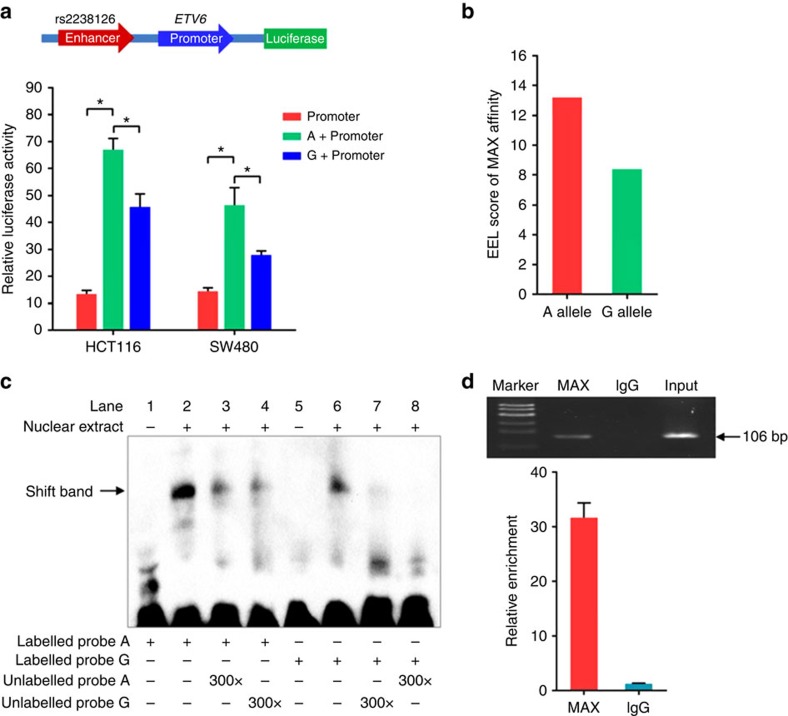
The rs2238126 alleles affect the activity of enhancer MAX at the 12p13.2 locus. (**a**) A putative enhancer region flanking rs2238126 (chr12:12,009,241-12,010,241) with A or G alleles was cloned upstream of the *ETV6* promoter-luciferase reporter vector. HCT116 and SW480 cells were transiently transfected with each of these constructs and assayed for luciferase activity after 24 h. The *P*-value was calculated with two-sided *t*-test. *P<0.001. (**b**) EEL analysis predicted the binding affinity of MAX to the rs2238126 alleles. (**c**) EMSA with biotin-labelled rs2238126 A or G probes and HCT116 nuclear extracts. Lanes 1 and 5 represent negative controls with probes only. The biotin-labelled rs2238126 A allele probe (lane 2) produced a much denser band of a specific DNA–protein complex (arrow) than the G allele probe (lane 6). The specific complex with rs2238126-labelled A probe can be partly competed by 300-fold unlabelled A probe (lane 3) or G probe (lane 4). The complex with the labelled G allele probe can be completely abolished by 300-fold unlabelled A probe (lane 8), but not G probe (lane 7). (**d**) ChIP and quantitative RT–PCR assays confirm that rs2238126 binds to MAX in HCT116 cells. Relative enrichment was calculated as a ratio of the signals from MAX or IgG to the signals from the input DNA.

The SNP rs2238126 lies in the intron of *ETV6.* RNA-Seq of 27 normal tissues demonstrated different expression levels of *ETV6* (Supplementary Fig. 7). Notably, moderate levels of *ETV6* were expressed in colon tissues relative to other normal tissues. Although a search of the ENCODE ChromHMM model from GM12878 lymphoblastoid cells revealed weak evidence of rs2238126 residing in a regulatory motif (Fig. 2), further examination of chromatin immunoprecipitation (ChIP)-sequencing (ChIP-seq) data suggested possible enhancer activities within the region encompassing rs2238126 in colorectal smooth muscle and HCT116 cells, on the basis of histone methylation marks and MAX binding (Supplementary Fig. 8). To determine the function of the rs2238126-containing enhancer in *ETV6* regulation, we constructed enhancer luciferase reporter vectors containing the rs2238126-centred region and the *ETV6* promoter. The rs2238126 A allele revealed a significantly increased enhancer activity compared with that of the G allele, and both alleles resulted in significantly stronger activation relative to the *ETV6* promoter, suggesting that the rs2238126-centred region acts as an enhancer (Fig. 3a). Enhancer Element Locator (EEL) prediction showed that rs2238126 directly affected a binding site for MAX (Fig. 3b). We also conducted an electrophoretic mobility shift assay (EMSA) to distinguish the differences in binding affinity between the rs2238126 A and G alleles to the transcription factor. The results confirmed that the A allele had a higher binding activity than the G allele (Fig. 3c). We further performed ChIP assay in HCT116 cells to verify that the rs2238126-containing region indeed bound the MAX *in vivo* (Fig. 3d).

We then performed an expression quantitative trait locus (eQTL) study to determine whether rs2238126 correlates with the mRNA expression levels of nearby genes (500 kb genomic region centred on rs2238126), using the Cancer Genome Atlas (TCGA) data of 434 colon adenocarcinoma tissues and 41 normal colon tissues. We found that rs2238126 was an eQTL for the *ETV6* (*P*_ANOVA_=3.46 × 10^−3^, Supplementary Fig. 9) and *BCL2L14* (*P*_ANOVA_=0.017) genes in colon tumour tissues but not in normal colon tissues (*ETV6*, *P*_ANOVA_=0.169; *BCL2L14*, *P*_ANOVA_=0.578). To further evaluate whether other SNPs at 12p13.2 act as eQTL for *ETV6*, we analysed the association between SNPs surrounding rs2238126 and the expression levels of *ETV6* (Supplementary Fig. 10). Our analysis showed that 13 SNPs were significantly associated with *ETV6* expression, of which rs2855708 was the most significant eQTL SNP (*P*_ANOVA_=5.34 × 10^−4^). However, this association was no longer statistically significant after adjusting for rs2238126.

### Functional analyses of *ETV6* in colorectal cancer

**Figure 4:**
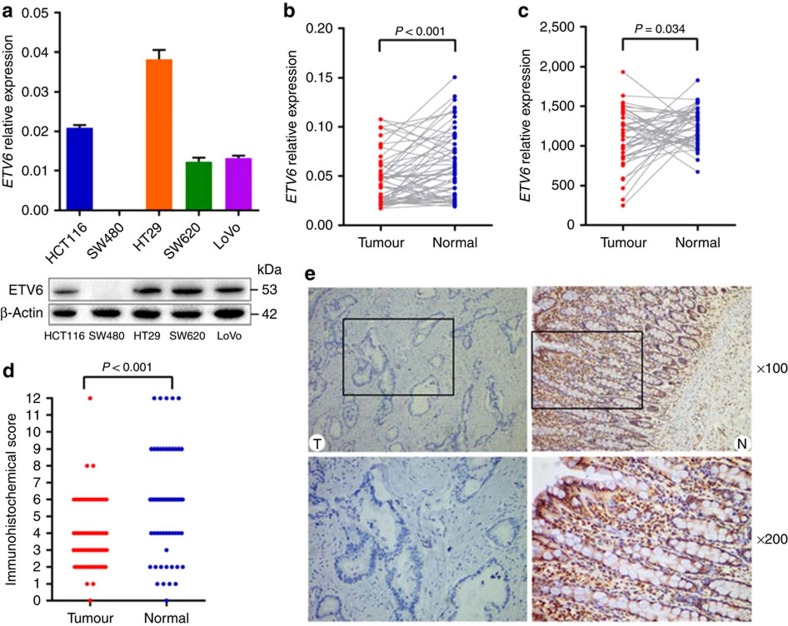
Expression of *ETV6* in human colorectal cancer cell lines and clinical specimens. (**a**) The *ETV6* mRNA (top) and protein (bottom) expression levels in five colorectal cancer cell lines. (**b**) The *ETV6* mRNA expression levels were estimated in 112 pairs of colorectal cancer tissues (T) and their adjacent normal tissues (N). The *P* value was calculated using the Wilcoxon matched-pairs signed-rank test. (**c**) The *ETV6* mRNA expression levels were analysed in paired colon tissues from 41 subjects from TCGA data. The *P*-values were determined using the paired *t*-test. (**d**) Semiquantitative analysis of the immunohistochemical staining intensity of 67 cancer tissues and corresponding adjacent normal tissues. (**e**) Representative immunohistochemical images of ETV6 protein expression in colorectal cancer tissue (left) and normal epithelial tissue (right). Top, × 100 magnification; bottom, × 200 magnification.

We measured the *ETV6* mRNA and protein expression levels in colorectal cancer cell lines and observed that *ETV6* expression was not detectable in the SW480 cell line (Fig. 4a). Next, we examined the mRNA expression levels of *ETV6* in 112 pairs of colorectal cancer tumours and their adjacent normal tissues and found significantly decreased *ETV6* expression in tumour tissues compared with their adjacent normal tissues (*P*_Wilcoxon_<0.001; Fig. 4b). This result was also supported by the data from the independent TCGA data, consisting of RNA-Seq of 41 paired colon tissues (*P*_*t*-test_=0.034; Fig. 4c). We randomly selected 67 pairs of colorectal cancer patients for immunohistochemical staining for ETV6 and found that ETV6 was highly expressed in the cytoplasm in tumours, whereas its expression in normal epithelial cells was primarily localized to the nuclei (Fig. 4e). We detected greater expression of ETV6 in adjacent normal colorectal tissues than in corresponding tumour tissues (*P*_Wilcoxon_<0.001; Fig. 4d).

To characterize the functional mechanism of *ETV6* in colorectal cancer, the *ETV6* overexpression or short hairpin RNA (shRNA) knockdown vectors were stably transfected into SW480, HCT116 and HT29 cells. As shown in Supplementary Fig. 11, overexpression of *ETV6* suppressed cellular growth, whereas knockdown of *ETV6* promoted proliferation. However, high or low *ETV6* expression did not induce statistically significant cell cycle changes (*P*_*t*-test_=0.115 in SW480 cells, *P*_*t*-test_=0.103 in HCT116 cells and *P*_*t*-test_=0.059 in HT29 cells for G1 phase). Similarly, the apoptosis of SW480, HCT116 and HT29 cells was not significantly altered by *ETV6* overexpression or knockdown (Supplementary Fig. 12). Consistent results were found after transiently transfecting SW480 cells with the *ETV6* overexpression vector (Supplementary Fig. 13).

### Cumulative effects of colorectal cancer susceptibility loci

Next, we assessed the cumulative effects of SNPs significantly associated with colorectal cancer risk. The risk alleles were normally distributed between the colorectal cancer cases and controls, and the distribution of these alleles was significantly different (*P*<0.001; Supplementary Fig. 14). Individuals carrying multiple risk alleles exhibited a gradual increase in the risk of colorectal cancer compared with those carrying 0–15 risk alleles (OR=1.41–5.09, *P*_trend_=2.34 × 10^−24^), suggesting a cumulative effect of associated genetic variants on colorectal cancer risk (Supplementary Table 8).

### Gene relationships across implicated loci (GRAIL) analysis

We performed a GRAIL analysis based on pathways previously defined in the literature to evaluate the connections between the genes located at all identified loci and the new susceptibility SNP rs2238126 (Supplementary Fig. 15). The identified connections showed that there was a higher-than-expected degree of connectivity, with a significance of *P*_GRAIL_<0.05 being observed for *TGFB1*, *SMAD7* and *BMP4*. rs2238126 in *ETV6* presented a weaker than expected connection with other genes reported in previous GWAS of colorectal cancer.

## Discussion

In this study, we used a three-stage genome-wide approach to identify associations between genetic variants and the risk of colorectal cancer. We found a new colorectal cancer-associated genetic locus rs2238126 at 12p13.2 in the Chinese population. The locus has not been identified in previous colorectal cancer GWAS. Our study findings suggest that genetic variants at 12p13.2 contribute to the development of colorectal cancer.

The SNP rs2238126 at 12p13.2 is located in intron 4 of *ETV6* (also known as *TEL*), an ETS family transcription factor that is essential for haematopoietic processes^20^,^21^. This ETS family gene has been identified as a potential prognostic marker of colorectal cancer invasiveness and metastasis^22^. Functional annotations revealed that rs2238126 mapped to a transcriptional enhancer-binding site for MAX. Reporter gene assay, EMSA and ChIP experiments on rs2238126 suggested that MAX is a regulatory enhancer transcription factor at the 12p13.2 locus. MAX has been characterized as a dimerization partner of MYC, which can induce cell-cycle progression and apoptosis^23^,^24^. MAX has multiple regulatory roles regarding histone decacetylases associated with activators and may participate in the tumorigenesis process in colorectal cancer^25^,^26^. Therefore, the contribution of rs2238126 to the development of colorectal cancer may result from the rs2238126 A allele preferentially binding MAX over the G allele.

The ETV6 protein contains two major domains, the ETS and HLH (helix–loop–helix) domains, which can be retained or lost at the site of the *ETV6* breakpoint. ETV6 is known to act as a strong transcriptional repressor in biological processes, including the regulation of cell growth and differentiation^27^,^28^,^29^. In this study, we found a higher protein expression level of ETV6 in normal colorectal tissues than in corresponding tumour tissues, which was consistent with the *ETV6* mRNA expression results. The eQTL analysis from TCGA data also revealed that rs2238126 was an eQTL for the *ETV6* and *BCL2L14* genes in colon tumour. In addition to *ETV6*, rs2238126 at 12p13.2 lies 214 kb upstream of *BCL2L14*, which belongs to the *BCL2* family and acts as anti- or pro-apoptotic regulators in a wide variety of cellular activities^30^. Therefore, the possibility that rs2238126 affects the *BCL2L14* gene and is related to colorectal cancer risk cannot be completely excluded. However, we failed to find an eQTL for the *ETV6* and *BCL2L14* genes in normal colon tissues. This result may be explained by sample size limitation or other factors, such as mutations or loss of heterozygosity in tumour tissues compared with normal tissues.

Previous studies have identified that upregulation of *ETV6* attenuates proliferation and suppresses Ras-induced transformation^31^. Consistently, our results revealed that the overexpression of *ETV6* dramatically inhibited cell growth. Based on these data, the *ETV6* gene can be considered to be a susceptibility gene for colorectal cancer, although the detailed molecular mechanisms underlying a regulatory role of *ETV6* in colorectal cancer remain to be further elucidated.

We compared the genotypes of rs2238126 among 14 populations from the 1000 Genome Project (Supplementary Table 9). The MAF of rs2238126 was found to be heterogeneous across these 14 populations (Supplementary Fig. 16). For example, the frequency of minor allele G was 0.477 in the Han Chinese population, whereas the frequency of the G allele was only 0.212 in the CEU population. The difference in MAFs may have an effect on patterns of LD for index association SNPs and causal SNPs between Asian and European individuals. Further studies among different ethnic groups are warranted to validate our findings.

We further selected all identified loci associated with colorectal cancer reported by previous GWAS for GRAIL analysis (Supplementary Fig. 15). GRAIL analysis revealed 19 regions with a significant score, including the strongest connections with TGF-β signalling pathway genes such as *TGFB1*, *SMAD7* and *BMP4*, thus suggesting a pivotal role of this pathway in colorectal cancer development. Notably, rs2238126 in *ETV6* was not related to previously implicated genes, thus supporting the role of *ETV6* as a potentially independent risk factor for colorectal cancer. These results suggest that genetic markers can be useful in risk prediction for colorectal cancer and that they are potential therapeutic targets.

**Figure 5:**
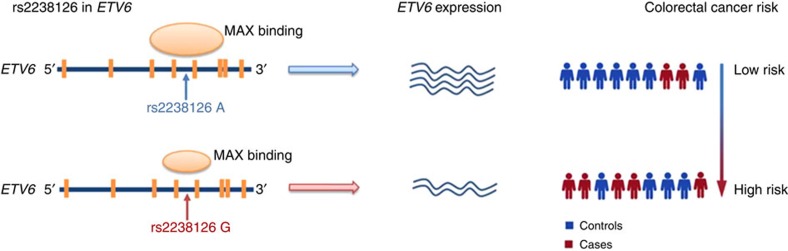
A schematic model of our findings. The *ETV6* gene expression is regulated by the SNP rs2238126. The rs2238126 G allele is associated with an increased risk of colorectal cancer because of decreased MAX binding, resulting in downregulating *ETV6* expression.

In summary, we identified a previously unknown colorectal cancer susceptibility locus in the *ETV6* gene at 12p13.2. The observed consistent association of rs2238126 in European populations provides convincing evidence for the novel colorectal cancer locus. The SNP rs2238126 G allele may attenuate the regulation of *ETV6*, which in turn is associated with increased risk of colorectal cancer, most likely by altering the binding affinity of transcriptional enhancer MAX (Fig. 5). Further functional studies are warranted to clarify the biological role of this region in the pathogenesis and aetiology of colorectal cancer.

## Methods

### Study subjects

All study subjects were Han Chinese population. We performed a three-stage GWAS for colorectal cancer. In the first GWAS stage, 1,049 colorectal cancer cases were enrolled from the Cancer Center of Nanjing Medical University and 1,315 controls were from the same districts of Nanjing beginning in September 2010 (Nanjing-1). The subjects in replication 1 included 855 colorectal cancer cases and 1,258 controls from the Nanjing First Hospital also from the same districts of Nanjing. The replication 2 sample sets were from six independent research centres in Wuhan (replication 2a, 805 cases and 1,200 controls), Guangzhou (replication 2b, 1,179 cases and 1,334 controls), Nanjing-3 (replication 2c, 612 cases and 1,188 controls), Xi'an (replication 2d, 643 cases and 384 controls), Hangzhou (replication 2e, 511 cases and 647 controls), Shenyang (replication 2f, 712 cases and 876 controls). The cases were diagnosed and histopathologically confirmed at the hospitals, and the controls were genetically unrelated to the cases. The controls in the GWAS stage were randomly selected from 25,000 subjects who participated in a community-based physical examination for noninfectious diseases in the same region. Additional controls in the replications were collected from those seeking medical care in local hospitals. Exclusions included participants who had been diagnosed with other colorectal disease, such as hereditary colorectal cancer syndromes. The participation rate of the eligible cases and controls exceeded 90%. Individuals who had smoked daily for more than one year were defined as smokers; otherwise, the subjects were considered as non-smokers. All of the subjects recruited for the three-stage study were evaluated with the same criteria^32^,^33^,^34^,^35^,^36^. We included 1,046 colorectal cancer cases and 1,076 controls from dbGaP (phs000779.v1.p1). All the subjects were from the Ontario Registry for Studies of Familial Colorectal Cancer^4^, which are part of the Genetics and Epidemiology Colorectal Cancer Consortium. The cases were confirmed incident colorectal cancer cases ages 20–74 years, residents of Ontario identified through comprehensive registry and diagnosed between July 1997 and June 2000. Population-based controls were randomly selected among Ontario, and matched by sex and 5-year age groups. Written informed consents were provided by all subjects. The study protocol was performed in accordance with the Institutional Review Board of Nanjing Medical University.

### SNP genotyping and data quality controls in the GWAS stage

Genomic DNA was derived from EDTA-venous blood by using the Qiagen Blood Kit (Qiagen). Genotyping for the GWAS stage was conducted using Illumina Human Omni ZhongHua Bead Chips for 900,015 SNPs. We used a uniform quality control protocol to filter the samples and the SNPs. Four subjects who failed to reach a genotype call rate of 95% were excluded. No sample was excluded because of sex discrepancies. An additional 27 samples were removed because of unexpected duplications or genetic relatedness. SNPs were excluded based on the SNPs (i) did not map on an autosomal chromosome; (ii) showed a MAF<0.05 in either the cases or the controls; (iii) displayed low call rate (< 95%) in all subjects or (iv) violated from Hardy–Weinberg equilibrium (*P*_*χ*2-test_<0.001) in the controls. We assessed the population stratification and outliers using a PCA method. In total, 30,456 common genotyped SNPs (MAF>0.25) with relatively low LD (*r*^2^<0.1) were used to estimate the outliers based on PCA (4 samples were identified).

### SNP selection and genotyping in the replication stages

To further confirm suggestive association in the GWAS stage, a subset of SNPs was selected for replication by using CLUMP analysis implemented in PLINK. The selected SNPs required *P*<1 × 10^−3^ in the GWAS stage and LD *r*^2^<0.5 between SNPs among our samples. In total, 53 SNPs were retained in the replication 1 stage.

Subjects in the replication 1 stage were genotyped using the Sequenom iPLEX MassARRAY assay. For quality control of genotyping, blinded duplicate samples from two subjects and two negative control (water) samples were included in each plate. In the replication 2 stage of rs2238126 analysis, the samples were genotyped by TaqMan assays using the ABI 7900HT Real-time PCR System (Applied Biosystems). Quality control samples were also used in the TaqMan assays, including one negative control (water) and two duplicates to which investigators were blinded. All of the primers for the Sequenom assay are presented in Supplementary Table 10. The genotyping cluster patterns for rs2238126 were examined to check high quality (Supplementary Fig. 17). Genotyping procedures were repeated by randomly selecting 5% of the participants, and the concordance rate was 100%.

### Imputation and regional association plotting

We imputed the non-genotyped SNPs based on the 1000 Genomes Project (Phase I, version 3, 1092 individuals) using IMPUTE2 (ref. 37). A series of filtering criteria for the imputed SNPs were implemented. Imputed SNPs were removed if they had (i) MAF<0.05; (ii) call rate<95% or (iv) Hardy–Weinberg equilibrium *P*<0.001. The association between genotype dosage data for imputed SNPs and colorectal cancer risk were analysed by the SNPTEST 2.5 program. Regional associations based on the results of the genotyped and imputed SNPs were plotted using LocusZoom 1.1.

### Functional annotation of rs2238126

We queried available ENCODE ChIP-seq data from colorectal smooth muscle and HCT116 cells for histone modification markers (H3K4me1, H3K4me3 and H3K27ac) and transcription regulator markers to determine whether rs2238126 fell within putative transcriptional regulatory elements. Transcription factor ChIP-seq data in HCT116 cells showed significant binding of MAX around rs2238126. We also used the EEL algorithm to investigate whether rs2238126 directly affected the MAX-binding site^38^. Further close examination of histone modifications was performed using the chromatin-state segmentation track (ChromHMM) from the GM12878 lymphoblastoid cells. The ENCODE data were visualized using the University of California Santa Cruz (UCSC) genome browser.

### Luciferase activity

The 1,000-bp containing rs2238126 A or G alleles of the enhancer sequence (chr12: 12,009,241-12,010,241) and *ETV6* promoter region (chr12: 11,801,788-11,802,787) were synthesized and cloned into the pGL3-basic vector (Promega) using the *Nhe*I and *Xho*I restriction sites. All constructs were confirmed by DNA sequencing.

For luciferase assays, HCT116 and SW480 cells were plated onto 24-well plates (3 × 10^5^ cells per well) and transfected with reporter plasmids using Lipofectamine 2000 (Invitrogen). As an internal standard, all plasmids were co-transfected with 10 ng pRL-SV40, which contained the *Renilla* luciferase gene. All transfections were performed in triplicate for each experiment. After transfection for 24 h, cells were collected and measured for the luciferase activity with a Dual-Luciferase Reporter Assay System (Promega). Relative luciferase activity was normalized to *Renilla* luciferase and statistically analysed with two-sided *t*-test.

### Electrophoretic mobility shift assay

Synthetic 3′ biotin-labelled 23-bp oligonucleotides and HCT116 cell nuclear extracts were incubated by using the LightShift Chemiluminescent EMSA Kit (Thermo Scientific). The oligonucleotide sequences are shown in Supplementary Table 10. For each gel shift sample (10 μl), a total of 1 μg nuclear extract was combined with 20 fmol labelled probes. For competition assays, unlabelled probes at 300-fold excess were added to the reaction before addition of labelled probes. Binding reactions were separated on a 4.5% polyacrylamide gel and detected by a chemiluminescent reaction with stabilized Streptavidin-horseradish peroxidase conjugate.

### ChIP assay

HCT116 cells were cross-linked with 1% formaldehyde at room temperature for 10 min. Nuclear extracts were sonicated to generate 200–1,000 bp chromatin fragments. The fragmented chromatin was immunoprecipitated using a ChIP assay kit (Upstate Biotechnology). The antibodies for the ChIP reaction were anti-MAX (ab53570, Abcam) and anti-rabbit IgG (2729, Cell Signaling Technology). Enrichment of the immunoprecipitation was assessed using gel electrophoresis and quantitative RT–PCR assays. The primers for RT–PCR are included in Supplementary Table 10. Quantification of enrichment was expressed as a ratio of MAX or IgG over the input control. Data points and error bars represent the mean and standard deviation, respectively, calculated from triplicates.

### Quantitative RT–PCR and eQTL analysis

Five colorectal cancer cell lines, HCT116, SW620, SW480, HT29 and LoVo, were maintained under standard conditions. These cell lines were purchased from Shanghai Institute of Biochemistry and Cell Biology, Chinese Academy of Sciences (Shanghai, China) within the past 2 years. Cell line authentication was conducted by China Center for Type Culture Collection (Wuhan, China) using short-tandem repeat profiling, and the results were compared with the American Type Culture Collections (ATCC) cell bank. No mycoplasma contamination was. All cell lines were tested and negative for mycoplasma contamination. Tumour tissue and paired adjacent normal tissue samples were collected from 112 subjects with colorectal cancer. The detailed information of patients is summarized in Supplementary Table 11. Total RNA was isolated from cultured cells and tissue samples using TRIzol (Invitrogen) and quantified by ultraviolet spectrometry. The relative mRNA expression level of *ETV6* and the internal control genes were detected using an ABI 7900 Real-Time PCR system (Applied Biosystems). The mRNA expression levels of *ACTINB*, *18sRNA*, *HRPT1*, *UBC* and *GAPDH* were examined to identify the most stably expressed housekeeping genes, and *ACTINB* and *GAPDH* were selected as endogenous controls using geNorm^39^. The geometric average of *ACTINB* and *GAPDH* expression was used as a reference to normalize the *ETV6* expression. The primer sets designed are presented in Supplementary Table 10.

The *ETV6* mRNA expression levels in normal tissues were measured using RNA-Seq of 27 different tissues from 95 human individuals, which are available at Array Express (accession number: E-MTAB-1733)^40^. The raw reads for each tissue were trimmed for low-quality ends. The average fragments per kilobase of exon model per million fragments mapped (FPKM) value of all individual samples was used to normalize mRNA expression.

Gene expression profiles were downloaded from TCGA project by RNA-Seq (level 3). In total, 434 colon adenocarcinoma tissues and 41 normal colon samples were included. To control for potential batch effects of mRNA expressions, a series of normalizations and corrections were applied, as described by Pickrell *et al*.^41^. Briefly, level 3 mRNA expression of each gene was log2 transformed if it was not normally distributed, and genes with zero values were removed. PCA was performed to correct gene expression, accounting for unmeasured confounders.

We also accessed TCGA individual level 2 SNP data from tissues and blood, which were genotyped with an Affymetrix Human Genome Wide SNP 6.0 array. SNPs from 500 kb flanking rs2238126 were used to impute genotypes based on the 1,000 Genomes Project using IMPUTE2. The analysis of variance (ANOVA) model was applied to assess the correlations between SNP genotypes and mRNA expression levels.

### Western blot and immunocytochemistry

Western blot assays were performed according to standard procedures. Cell lysates were extracted using a detergent lysis buffer supplemented with a protease inhibitor. Equal amounts (40 μg) of protein samples were subjected to SDS–polyacrylamide gel electrophoresis and transferred to semi-dry blotted polyvinylidene difluoride membrane (Millipore). The primary antibodies used for the protein analyses were monoclonal rabbit anti-ETV6, 1:1,000 (ab151698, Abcam); and rabbit anti-β-actin, 1:1,000 (13E5, Cell Signaling Technology). The secondary antibody used for protein analyses was anti-rabbit HRP, 1:1,000 (BS13278, Bioworld Technology). The immune complexes were detected by enhanced chemiluminescence (Cell Signaling Technology). Uncropped blots are shown in Supplementary Fig. 20.

In total, 67 paired surgical colorectal cancer specimens were fixed in formalin, routinely processed and embedded in paraffin. The primary antibody applied for immunohistochemical detection of ETV6 protein expression was the same as that used for western blot. Two experienced pathologists scored the staining results in a blinded manner. The immunostaining intensity was scored as 0 (negative), 1 (weak), 2 (moderate) or 3 (strong; Supplementary Fig. 18), and the percentage of stained cells was scored semiquantitatively as 1 (0–25%), 2 (26–50%), 3 (51–75%) or 4 (76–100%). Multiplication of the intensity and percentage scores resulted in a score ranging from 0 to 12 for each tissue. The difference of scores was assessed using the Wilcoxon matched-pairs signed-rank test.

### Construction and transfection of overexpression and knockdown of *ETV6*

For the stable overexpression and knockdown of *ETV6* in colorectal cancer cells, one *ETV6* cDNA and three independent shRNAs were designed and cloned into the GV358 and GV248 lentivirus vector (GeneChem), respectively. The plasmid sequences were confirmed by DNA sequence analysis. The sequences of three shRNAs are shown in Supplementary Table 10. The vectors were transfected using the Polybrene and Enhanced Infection Solution according to the manufacturer's protocol (GeneChem). The cells were infected with lentivirus at a multiplicity of infection of 10. The transfected cells were further selected with 2 μg ml^−1^ puromycin for 2 weeks. The stable effect of *ETV6* overexpression and knockdown was determined by quantitative RT–PCR and western blot (Supplementary Fig. 19).

For the transiently transfected SW480 cells, the *ETV6* overexpression vector was constructed and cloned into the GV230 vector (GeneChem). The plasmid sequences were confirmed by sequencing. For transient transfection, Lipofectamine 2000 transfection reagent (Invitrogen) was used according to the manufacturer's protocol.

### Cell proliferation and cell death assays

For cell proliferation analysis, the proliferation of colorectal cancer cells was evaluated using a Cell Counting Kit-8 (CCK-8; Dojindo) at various time intervals. Cell growth was represented by the absorbance at an optical density of 450 nm using an Infinite M200 spectrophotometer (Tecan). For the cell cycle assay, cells were fixed with 75% ethyl alcohol, stained with propidium iodide and the assay was performed using a FACS Calibur flow cytometer (Beckman Coulter). For apoptosis detection, cells were collected and stained using an Annexin V-FITC apoptosis detection kit (Invitrogen), and flow cytometry was used to detect the percentage of apoptotic cells. All experiments were independently performed at least three times and data were expressed as a mean and standard deviation. Statistical comparisons were analysed with two-sided *t*-test.

### Statistical analysis

The association between each SNP and colorectal cancer risk was evaluated under an additive model with adjustment for eigenvectors, age and sex using PLINK1.07. The population structure was estimated by a PCA using EIGENSOFT 5.0.1, and the Manhattan plot based on the −log10 *P* was created by using R 2.15.0. The first two eigenvectors for each individual were plotted. For the combined analysis, a meta-analysis of the OR weighted based on the 95% confidence interval was conducted under a fixed-effects model. The measure of heterogeneity was tested using Cochran's Q statistics and *I*^2^. We used the Haploview 4.2 to visualize the LD structure of chromosome 12p13.2. The biological relationships between the genes within the GWAS-reported loci were quantified using GRAIL^42^. Alternatively, the analyses were performed using SAS 9.2 (SAS Institute) or Stata 10.0 (StataCorp LP).

### Data availability

The genotyping data has been deposited in the Dryad Digital Repository ( DOI: 10.5061/dryad.7dj7t) (ref. 43).

## Additional information

**How to cite this article:** Wang, M. *et al*. Common genetic variation in *ETV6* is associated with colorectal cancer susceptibility. *Nat. Commun.* 7:11478 doi: 10.1038/ncomms11478 (2016).

## Supplementary Material

Supplementary Information Supplementary Figures 1-20 and Supplementary Tables 1-11.

## Figures and Tables

**Figure 1:**
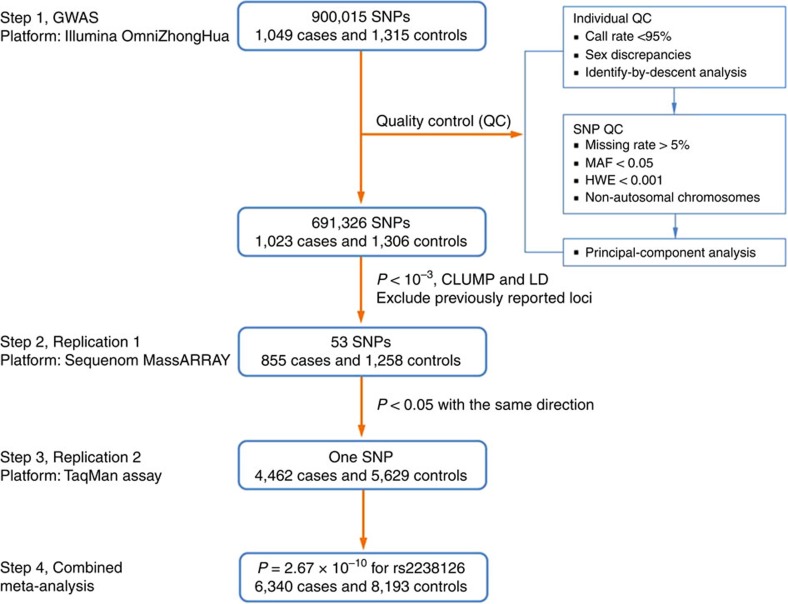
Summary of the study design and the results. A three-stage GWAS involving 1,049 cases and 1,315 controls was conducted in stage 1 and the most significant SNPs were followed up in two stages of replication including 5,317 cases and 6,887 controls.

**Table 1:** Association of rs2238126 at 12p13.2 associated with colorectal cancer among individuals from eight Chinese study centres.

**SNP**	Allele*	**Studygroup**	**Population**	**Samplesize**		**Genotypes**†		**MAF**‡		**OR(95%CI)**§	***P*-value**§	***P***_**het**_||	***I***^**2**^
				**Cases**	**Controls**	**Cases**	**Controls**	**Cases**	**Controls**				
rs2238126	A/G	GWAS	Nanjing-1	1,023	1,306	280/516/227	304/629/373	0.526	0.474	1.25(1.10–1.43)	7.41 × 10^−4^		
		Replication 1	Nanjing-2	855	1,258	228/425/189	292/615/347	0.523	0.478	1.20(1.06–1.36)	4.46 × 10^−3^		
		Replication 2											
		Replication 2a	Wuhan	805	1,200	206/399/200	283/585/332	0.504	0.480	1.10(0.97–1.25)	0.137		
		Replication 2b	Guangzhou	1,179	1,334	300/620/259	287/682/365	0.517	0.471	1.26(1.11–1.43)	2.57 × 10^−4^		
		Replication 2c	Nanjing-3	612	1,188	156/309/147	293/584/311	0.507	0.477	1.13(0.98–1.29)	0.093		
		Replication2d	Xi'an	643	384	164/325/154	92/183/109	0.508	0.478	1.13(0.95–1.35)	0.180		
		Replication 2e	Hangzhou	511	647	146/246/119	154/314/179	0.526	0.481	1.19(1.02–1.40)	0.032		
		Replication 2f	Shenyang	712	876	180/358/174	200/443/233	0.504	0.481	1.08(0.93–1.25)	0.336		
		Replication 2 combined		4,462	5,629			0.511	0.477	1.15(1.08–1.21)	2.72 × 10^−6^	0.590	0
		All combined¶		6,340	8,193			0.515	0.477	1.17(1.11–1.23)	2.67 × 10^−10^	0.626	0

CI, confidence interval; GWAS, genome-wide association study; MAF, minor allele frequency; OR, odds ratio; SNP, single-nucleotide polymorphism.

^*^Major/minor allele.

^†^The distribution of GG, GA and AA genotypes.

^‡^MAF of G allele.

^§^OR, 95% CI and the corresponding *P*-values were derived from logistic regression analysis under an additive model with adjustment for top eigen, age and sex, where appropriate.

^||^*P* value for the heterogeneity.

^¶^GWAS and replication stages were combined by meta-analysis under a fixed-effects model.

**Table 2:** Association of colorectal cancer risk with rs2238126 at 12p13.2 in individuals of European and Asian populations combined.

**SNP**	Allele*	**Study**	**Population**	**Sample size**		**MAF**†		**OR (95% CI)**‡	***P*-value**‡
				**Cases**	**Controls**	**Cases**	**Controls**		
rs2238126	A/G	OFCCR	European	1,046	1,076	0.179	0.155	1.19 (1.01–2.12)	0.034
		This study	Asian	6,340	8,193	0.515	0.477	1.17 (1.11–1.23)	2.67 × 10^−10^
		Meta-analysis§						1.17 (1.12–1.23)	2.79 × 10^−11^

CI, confidence interval; GWAS, genome-wide association study; MAF, minor allele frequency; OFCCR, ontario registry for studies of familial colorectal cancer; OR, odds ratio; SNP, single-nucleotide polymorphism.

^*^Major/minor allele.

^†^MAF of G allele.

^‡^Additive model.

^§^Results were combined by meta-analysis using a fixed-effects model (*P*_heterogeneity_=0.853, *I*^2^=0).
