# Supplementary material for: Common genetic variation in ETV6 is associated with colorectal cancer susceptibility
Source: Nat Commun. 2016 May 5;7:11478. doi: 10.1038/ncomms11478 (PMC4858728; doi:10.1038/ncomms11478)
Supplement: Supplementary Information — Supplementary Figures 1-20 and Supplementary Tables 1-11. [file ncomms11478-s1.pdf]

Supplementary Figure 1. Principle component analysis based on the GWAS subjects and the HapMap Phase 2 populations. (A) Distributions of all subjects in the GWAS stage and four HapMap populations; (B) Distributions of the cases and controls in the GWAS stage.

A

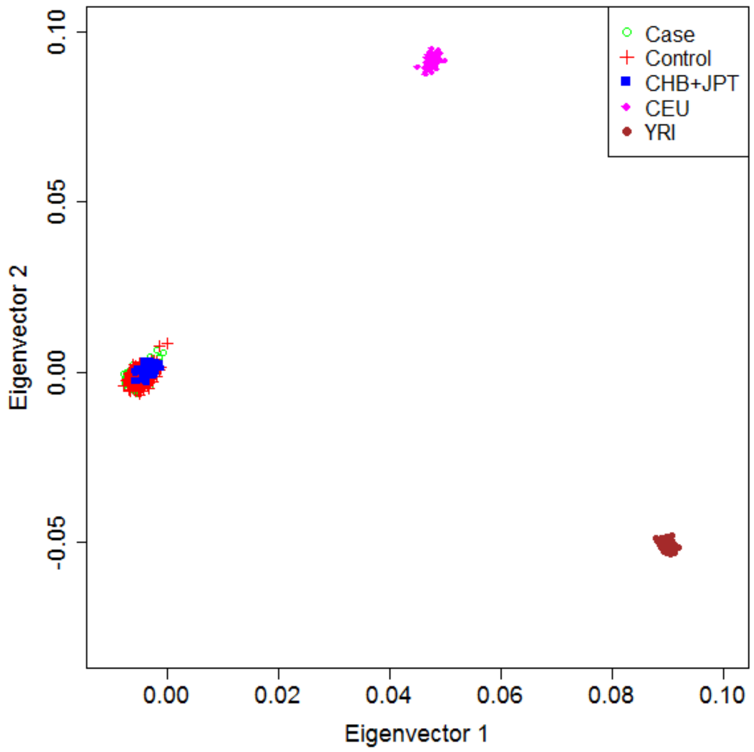

B

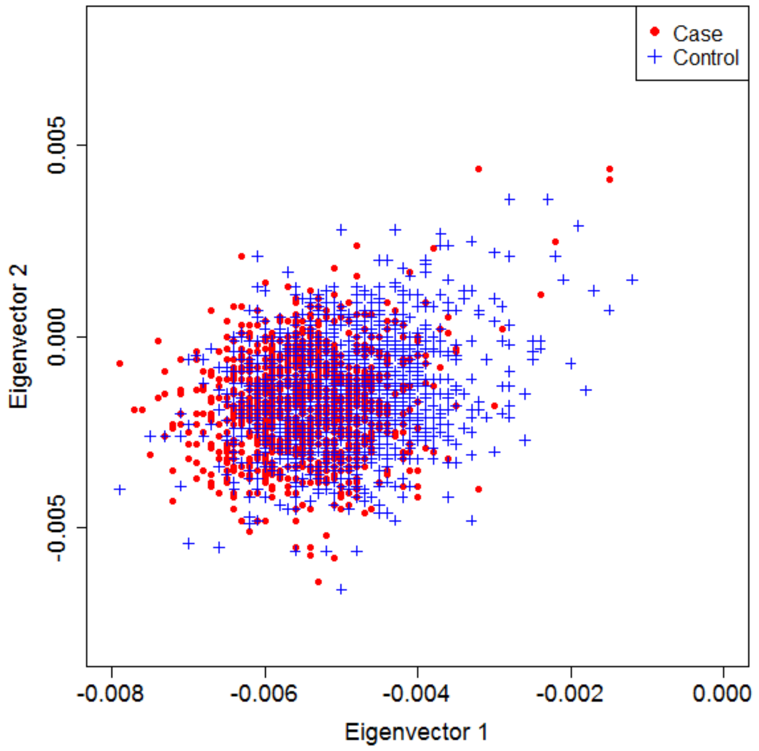

Supplementary Figure 2. Quantile-quantile plot and genomic inflation factor λ for associations with colorectal cancer risk.

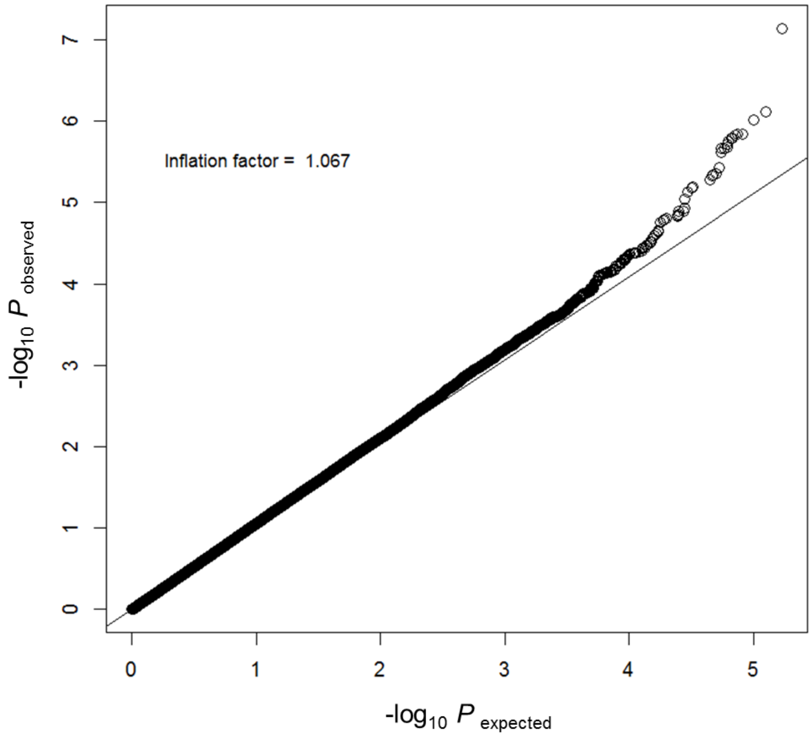

Supplementary Figure 3. Manhattan plot of the genome-wide association with colorectal cancer in the Chinese population. The scatter plot of the *P* values in -$\log_{10}$ scale represents the association results from additive model in 1,023 cases and 1,306 controls. The *x* axis represents the chromosomal and SNP position.

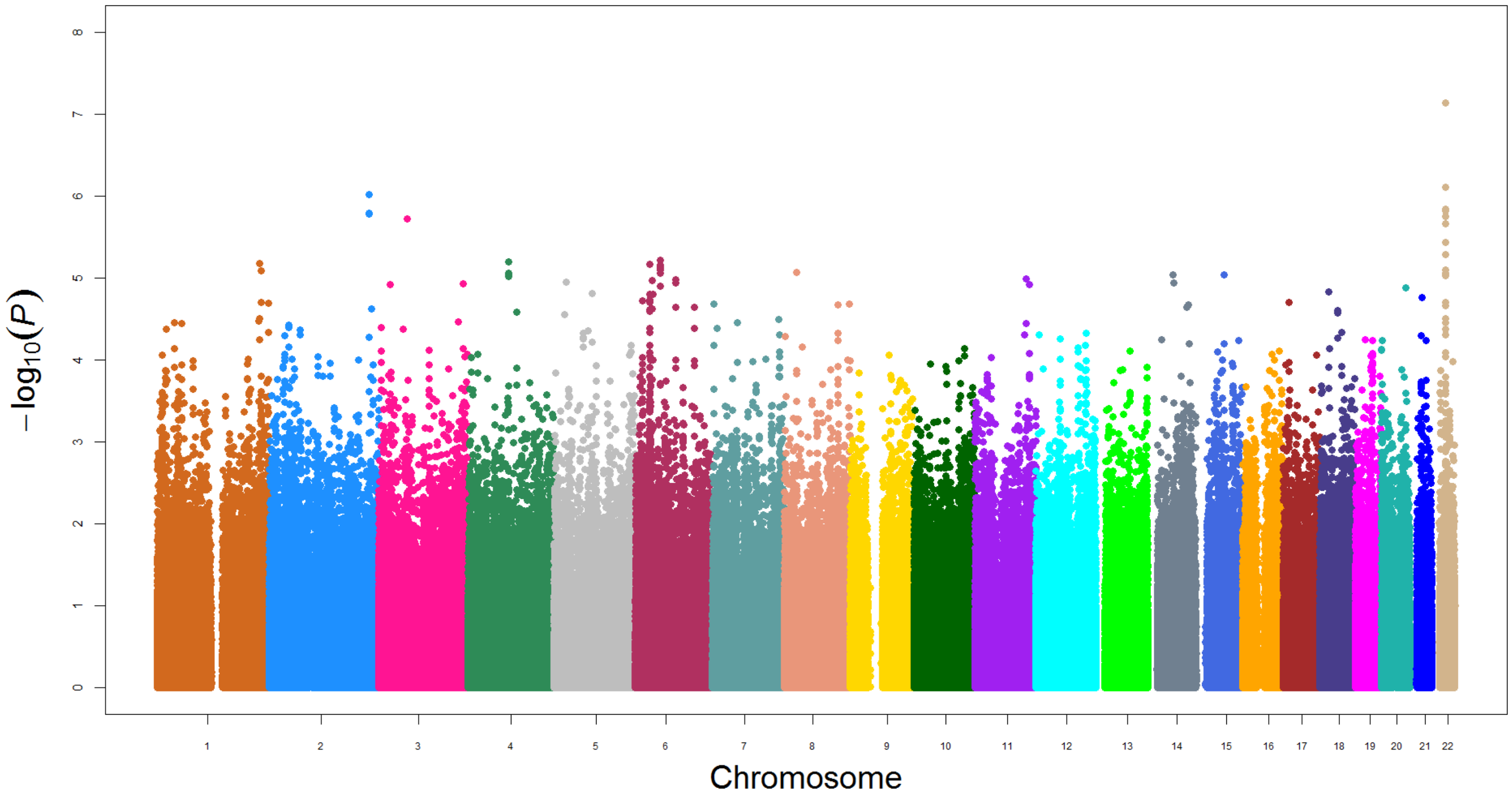

Supplementary Figure 4. Forest plot of the meta-analyses of the association between rs2238126 and colorectal cancer risk in 8 Chinese study centers. The horizontal axis plots of the OR and the 95% CI. The diamond represents the pooled OR.

Stage, population	Cases/controls	OR (95% CI)
GWAS, Nanjing-1	1023/1306	1.25 (1.10-1.43)
Replication1, Nanjing-2	855/1258	1.20 (1.06-1.36)
Replication 2a, Wuhan	805/1200	1.10 (0.97-1.25)
Replication 2b, Guangzhou	1179/1334	1.26 (1.11-1.43)
Replication 2c, Nanjing-3	612/1188	1.13 (0.98-1.29)
Replication 2d, Xi'an	643/384	1.13 (0.95-1.35)
Replication 2e, Hangzhou	511/647	1.19 (1.02-1.40)
Replication 2f, Shenyang	712/876	1.08 (0.93-1.25)
**Combined**	**6340/8193**	**1.17 (1.11-1.23)**

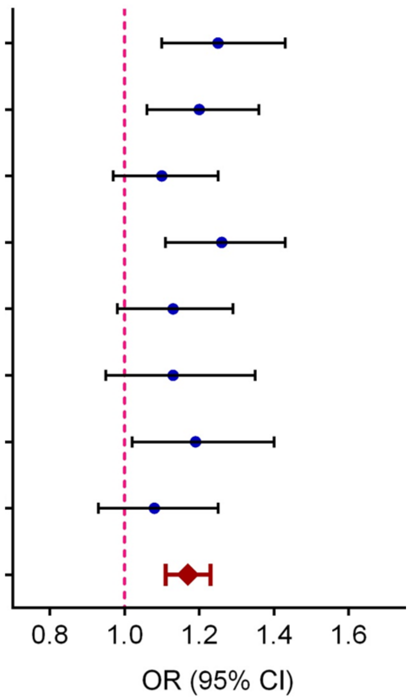

I^2 = 0, P =0.626 for the heterogeneity

Supplementary Figure 5. Stratification analyses of the associations between rs2238126 and colorectal cancer risk in the combined case-control subjects. Each point and horizontal line represents the OR and 95% CI calculated using the additive model. The *P* values were calculated using heterogeneity tests.

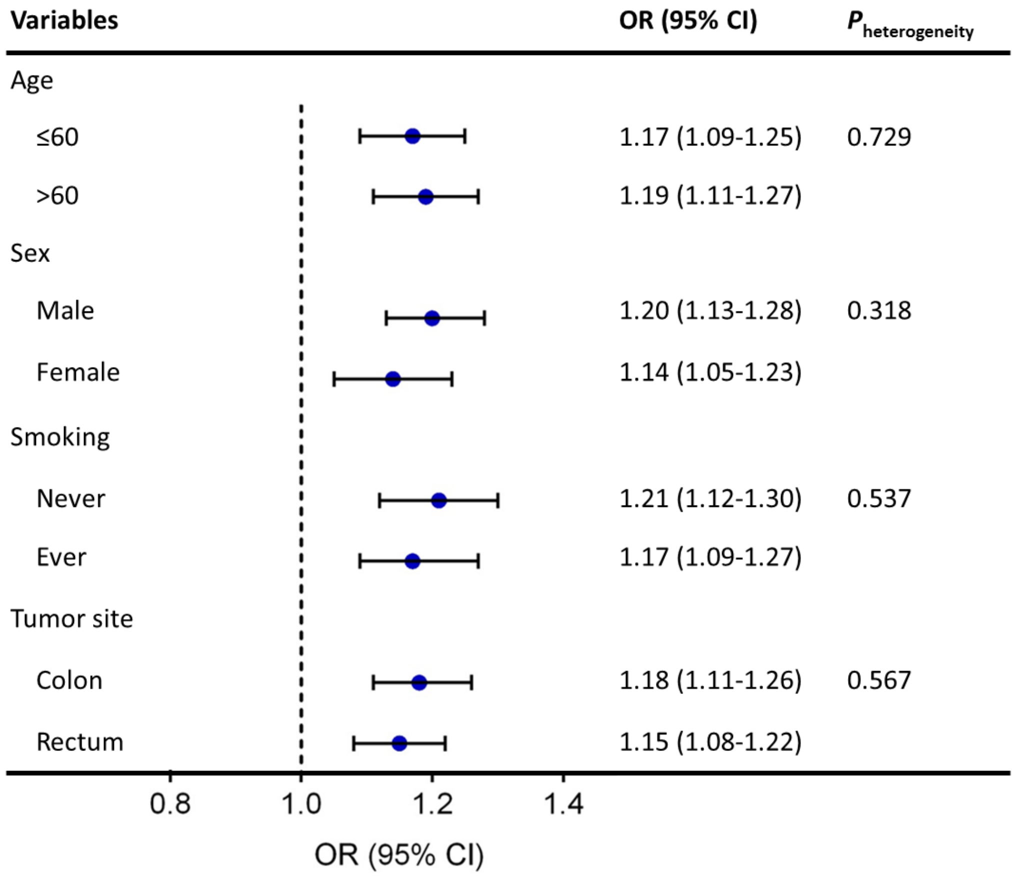

Supplementary Figure 6. The association between the rs2238126 genotypes and age at diagnosis. The numbers of the AA, AG, and GG genotypes were 1439, 3258, and 1630, respectively. The error bars show mean ± SD.

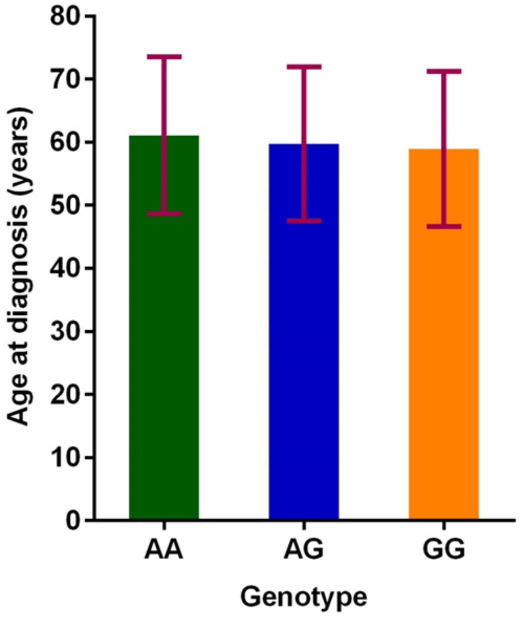

Genotype	N (%)	Mean ± SD (years)
AA	1439 (22.7%)	61.1 ± 12.5
AG	3258 (51.5%)	59.7 ± 12.2
GG	1630 (25.8%)	58.9 ± 12.3

Supplementary Figure 7. The *ETV6* mRNA expression levels in normal tissues.

Tissue-specificity mRNA expression analysis was performed using RNA-Seq of 27 different tissues from 95 human individuals (E-MTAB-1733). The relative mRNA expression levels were normalized to the FPKM value. The orange bar represents the *ETV6* expression level in human colon tissue.

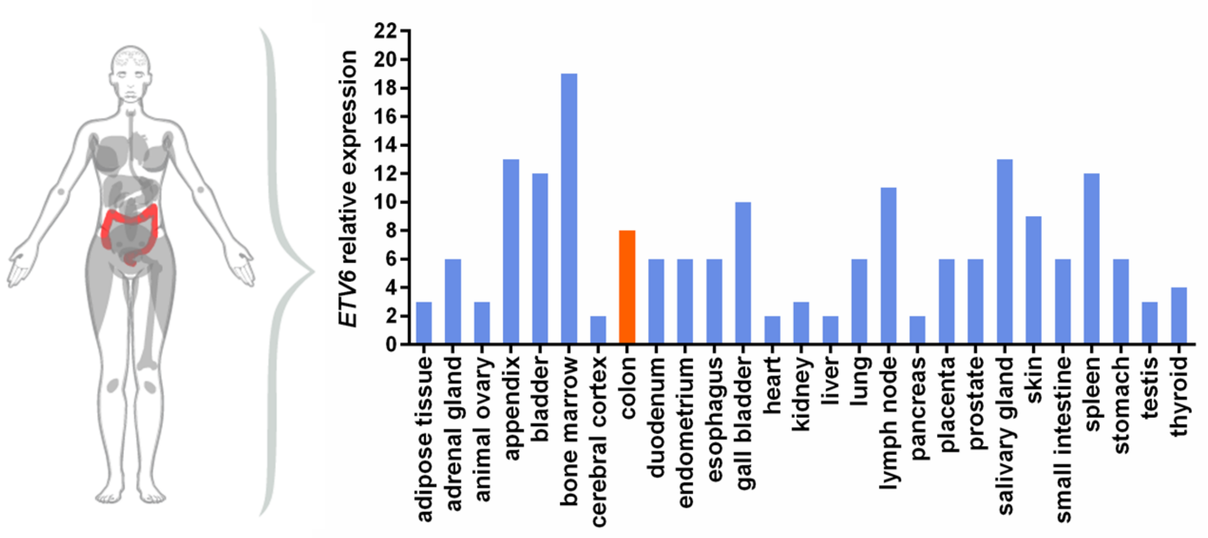

Supplementary Figure 8. Functional annotation at 12p13.2 in a region 500 kb upstream and downstream of rs2238126 based on the ENCODE Project. Histone modification marks (H3K4me1, H3K4me3, and H3K27ac) were obtained from ChIP-seq data of colorectal smooth muscle and HCT116 cells, whereas the transcriptional factor MAX was predicted from HCT116 cells. The red line represents the position of rs2238126.

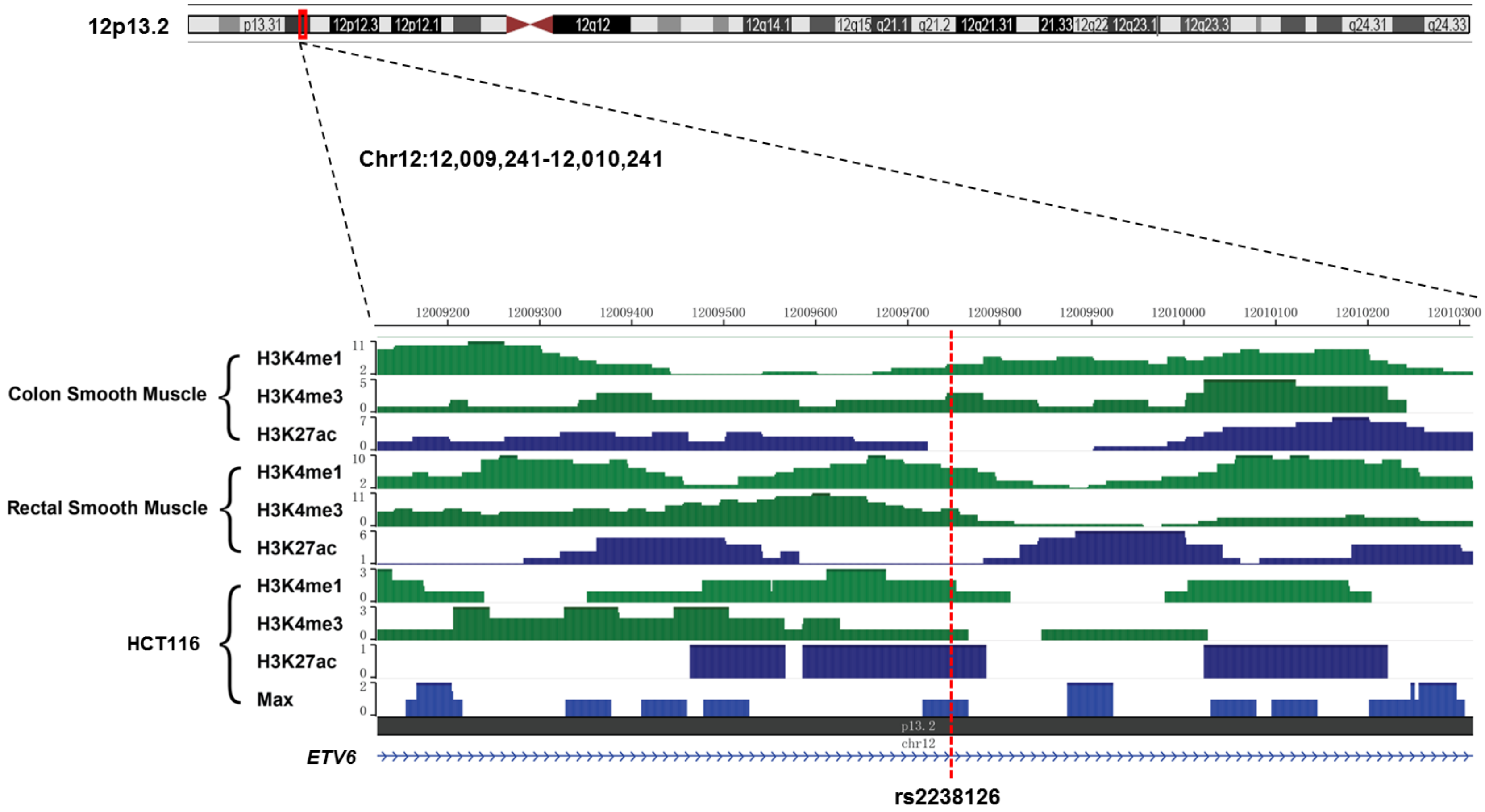

Supplementary Figure 9. Association between rs2238126 genotypes and *ETV6* mRNA expression levels in TCGA data of (A) 434 colon adenocarcinoma tissues and (B) 41 normal colon tissues**.** The *ETV6* mRNA expression levels were log2 transformed. The *P* value was calculated using ANOVA model.

A

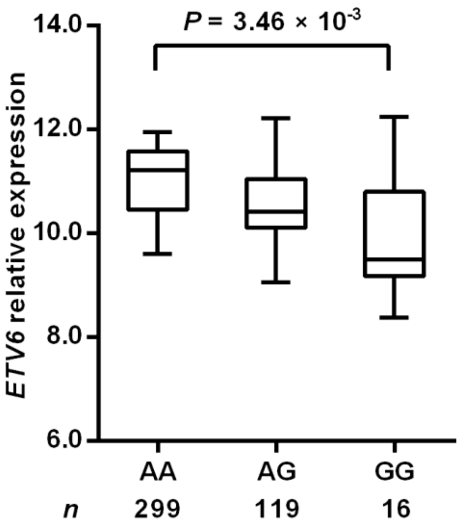

B

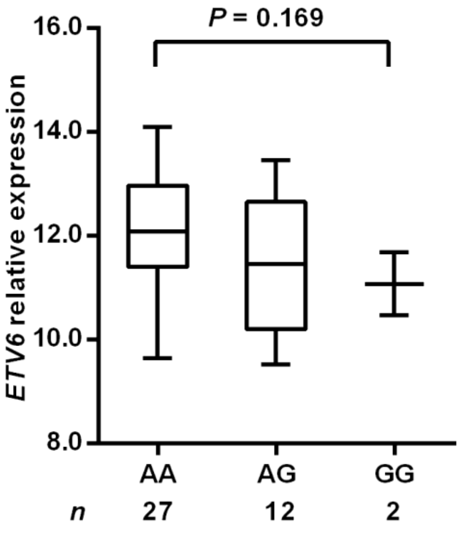

Supplementary Figure 10. Regional plot of eQTL association results of rs2238126 at 12p13.2 with *ETV6* expressions in TCGA data of 434 colon adenocarcinoma tissues. The association results (-$\log_{10}$ *P*) are shown for SNPs in the region 400 kb upstream and downstream of rs2238126. The relationship between SNP genotypes and the *ETV6* mRNA expression levels was evaluated using ANOVA model.

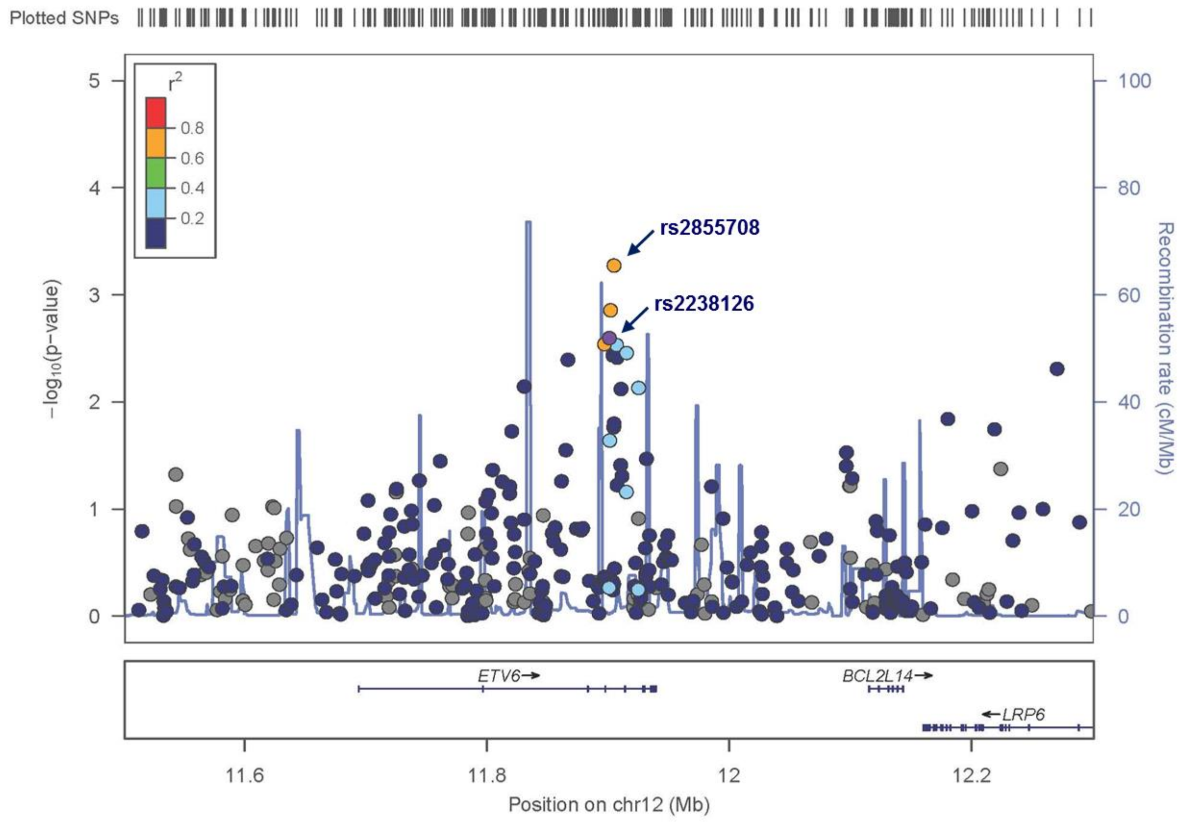

Supplementary Figure 11. Roles of *ETV6* in regulating colorectal cancer cell growth and cell cycle. (A) Cell proliferation of SW480, HCT116 and HT29 cells was measured by the CCK8 assay (OD450 absorbance). *, *P* < 0.001. (B) Representative results of the cell cycle of SW480, HCT116 and HT29 cells transfected with the *ETV6* overexpression or knockdown vectors. The cell cycle was analyzed by flow cytometry. Experiments were performed in triplicate and repeated three times. The data are expressed as the means and SD from three replicate samples, and the statistical comparisons were analyzed by two-sided *t*-test.

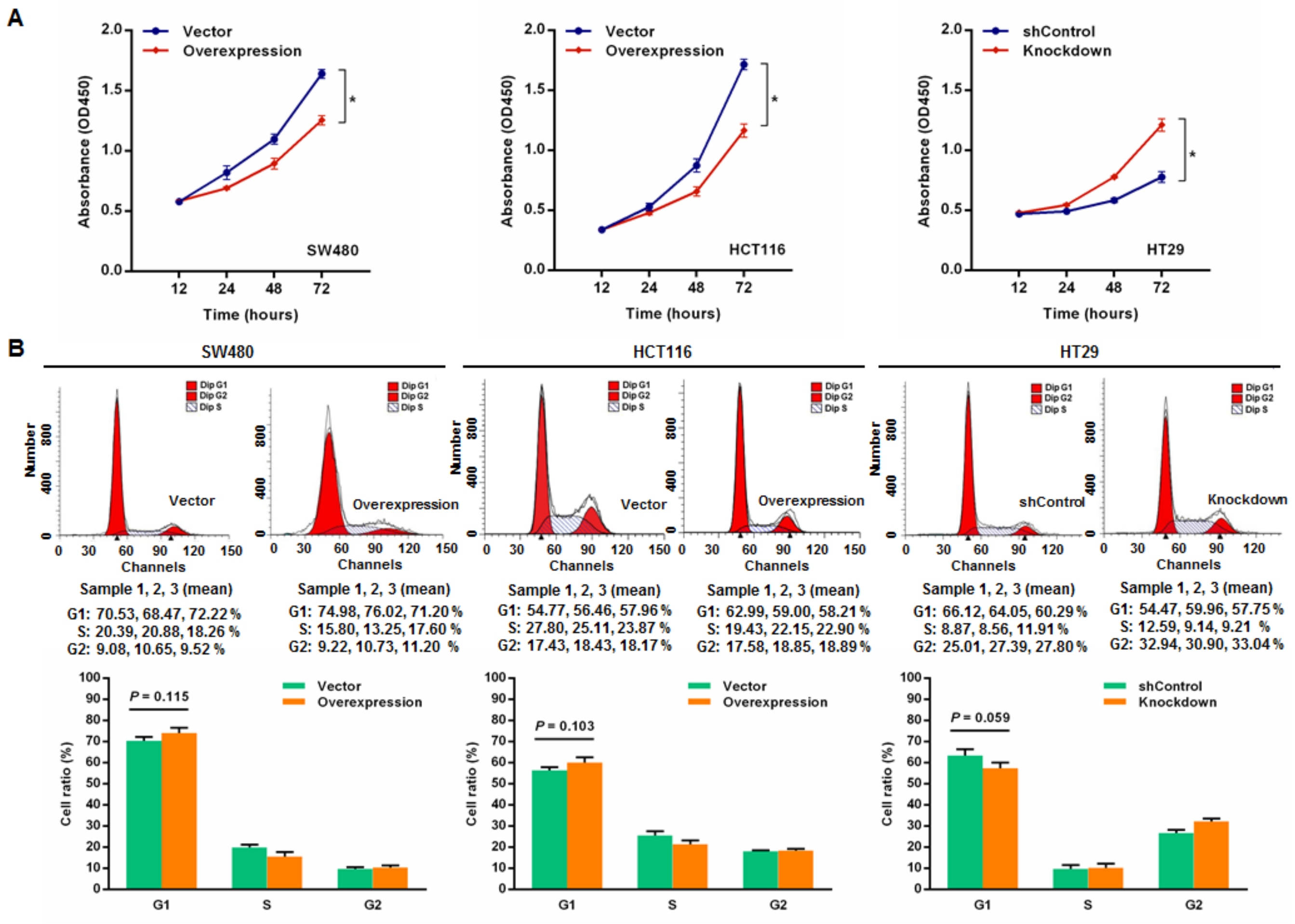

Supplementary Figure 12. Effects of *ETV6* overexpression on the apoptosis of SW480, HCT116 and HT29 cells. The cells transfected with the *ETV6* overexpression or knockdown vectors were stained and detected by flow cytometry. The apoptosis rates are expressed as the means and SD of triplicate samples. The *P* value was calculated by two-sided *t*-test. LR, early apoptotic cells; UR, terminal apoptotic cells.

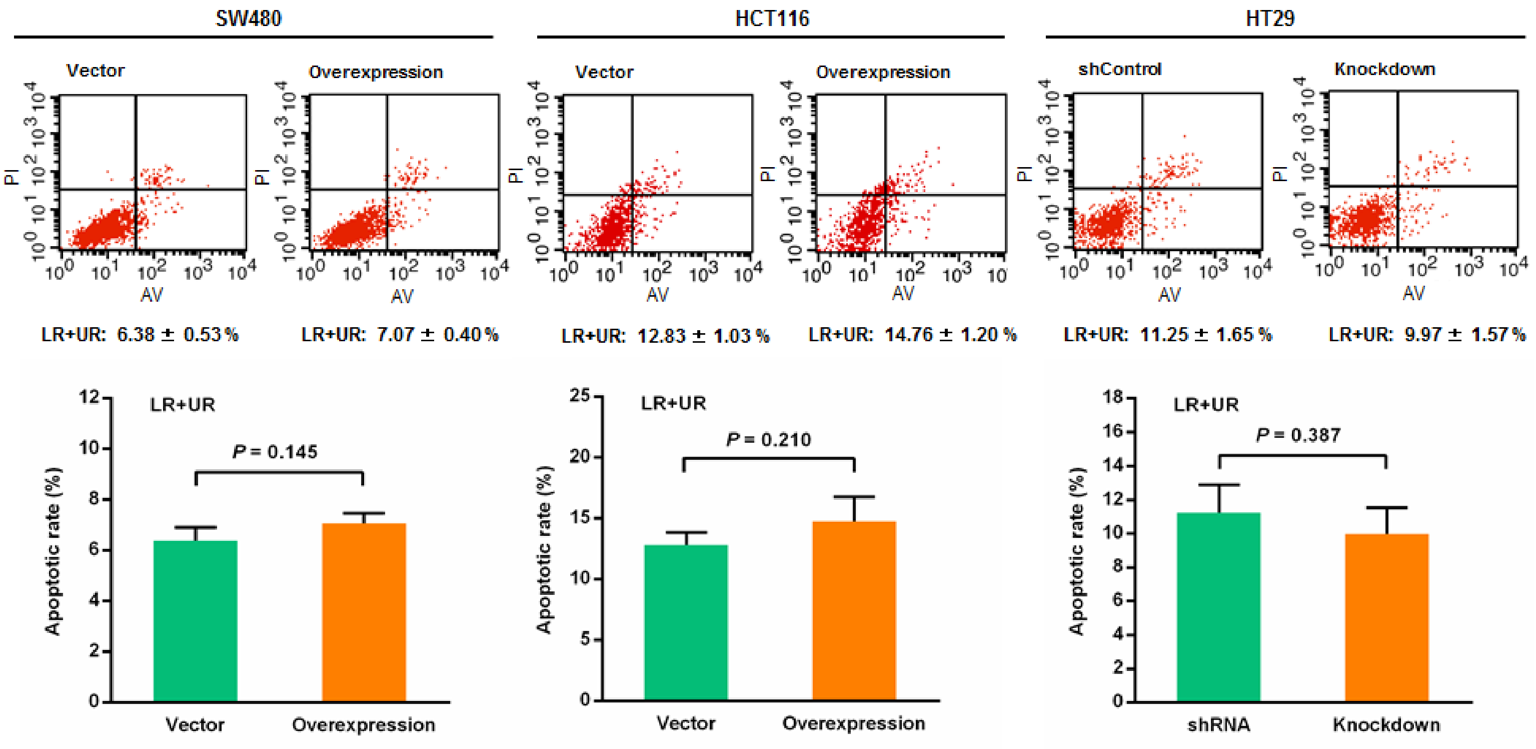

Supplementary Figure 13. Transiently transfected with overexpression of *ETV6* in regulating cell growth, cell cycle, apoptosis of SW480 cells. (A) Cell proliferation of SW480 cells was measured by the CCK8 assay (OD450 absorbance). *, $P < 0.001$. (B) The cell cycle of SW480 cells was analyzed by flow cytometry. (C) Representative results of the apoptosis of SW480 cells. (D) The apoptosis rates of SW480 cells were stained and detected by flow cytometry. The cell growth, cell cycle, and apoptosis rates are expressed as the means and SD of triplicate samples, and these experiments were repeated three times using the same methods. The statistical comparisons were analyzed by two-sided t-test. LR, early apoptotic cells; UR, terminal apoptotic cells.

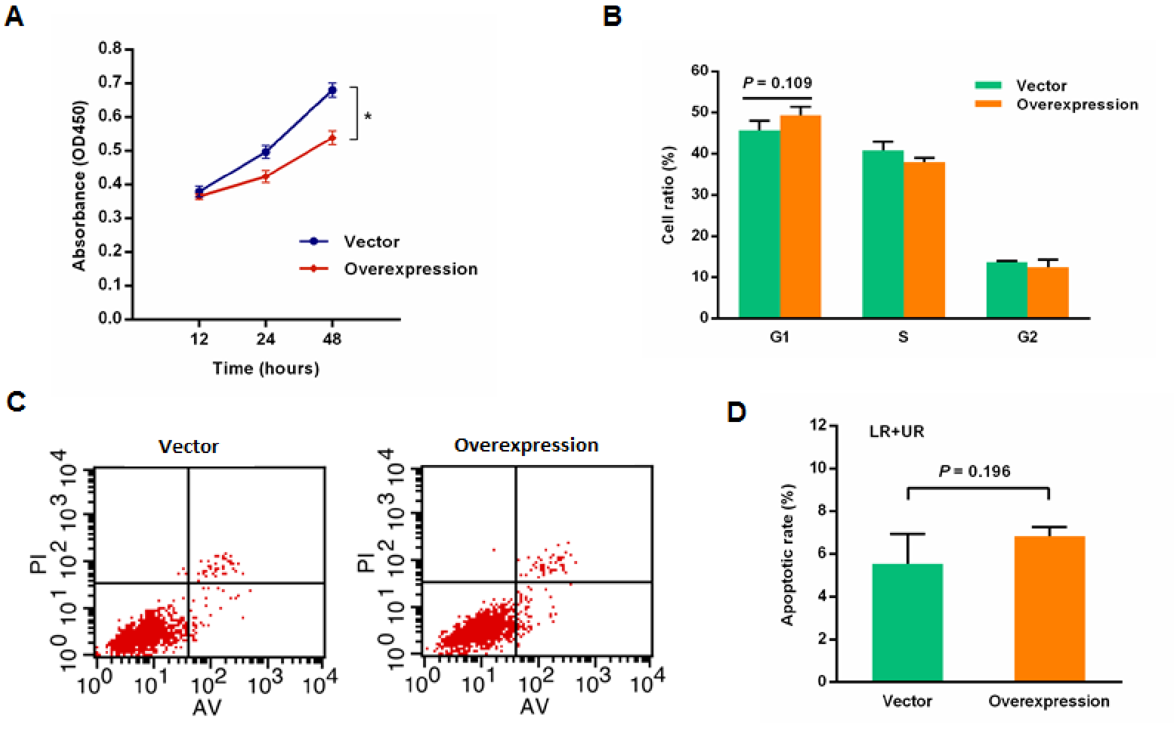

Supplementary Figure 14. Cumulative effect of rs2238126 and previously reported SNPs on colorectal cancer risk in the GWAS stage. (A) Distribution of the risk alleles between the colorectal cancer cases (orange) and the controls (blue). (B) Plot displaying the increasing OR for colorectal cancer with an increasing number of risk alleles. Each point and vertical line represents the OR and the 95% CI, respectively.

A

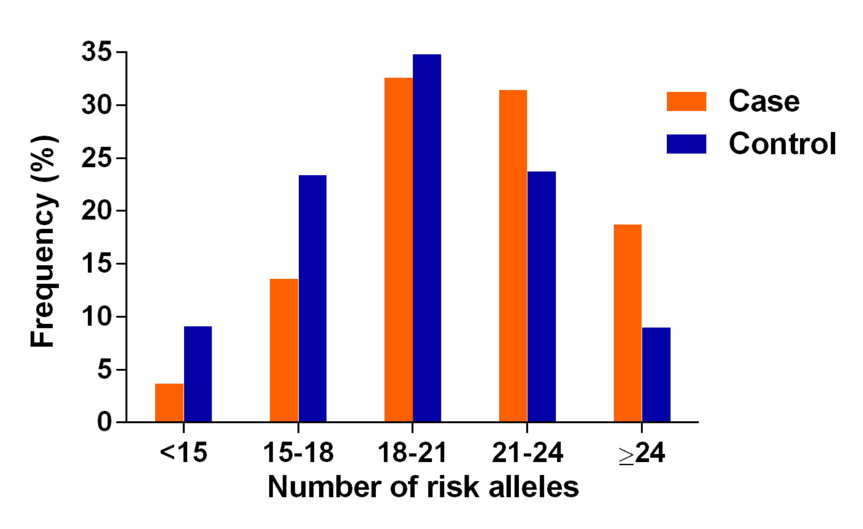

B

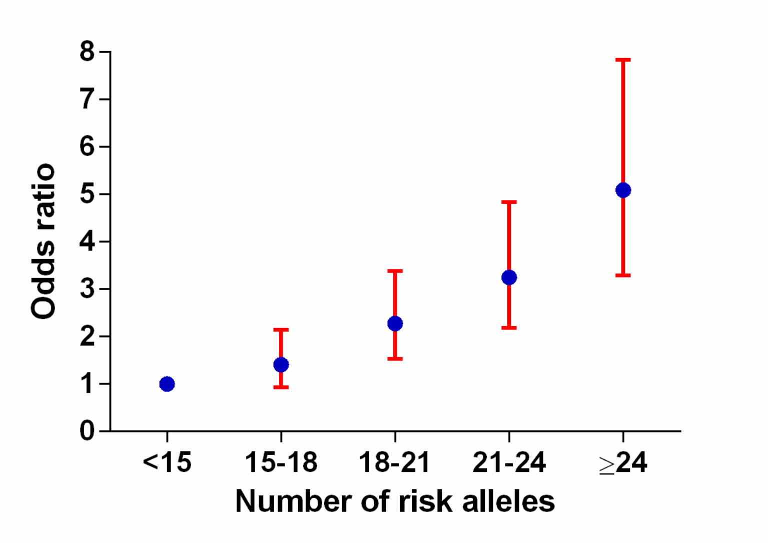

Supplementary Figure 15. GRAIL analysis was performed to identify the GWAS-reported SNPs in highly related genes. The functional similarity between the genes was measured using a text-mining method on the PubMed database before 2006. The thickness of the red lines indicates the strength of the literature-based connectivity between the genes. The colorectal cancer-associated loci are along the outer circle, and the internal circle contains the connected genes. The SNP rs2238126 and the *ETV6* gene, shown in red, were derived from our GWAS of colorectal cancer.

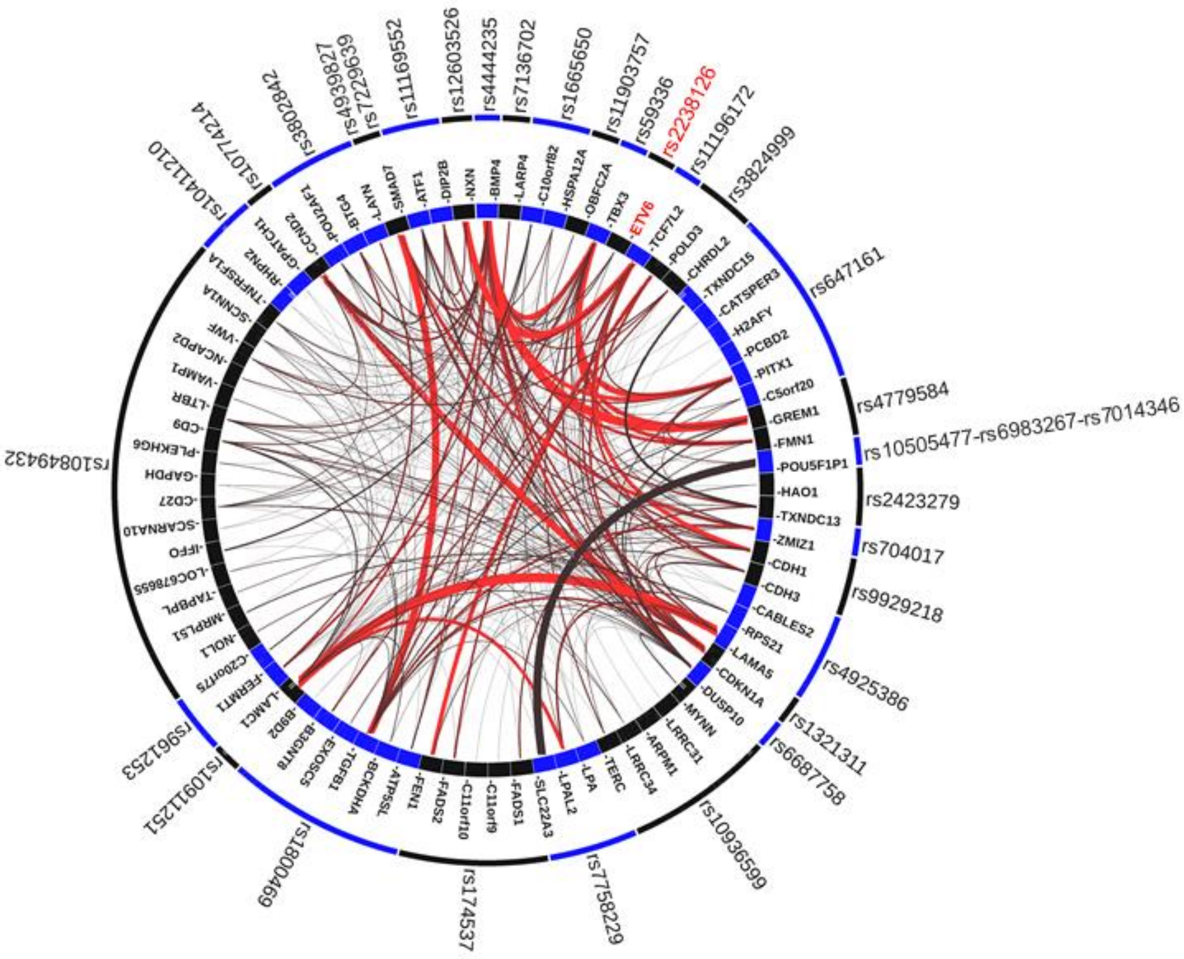

Supplementary Figure 16. The rs2238126 allele frequency in diverse population from the 1000 Genome Project. Population descriptions, ASW, African Ancestry in Southwest USA; CEU, (CEPH) with Northern and Western European ancestry; CHB , Han Chinese in Beijing, China; CHS, Han Chinese South; CLM, Colombian in Medellin, Colombia; FIN, Finnish in Finland; GBR, British in England and Scotland ; IBS, Iberian populations in Spain; JPT, Japanese in Tokyo, Japan; LWK, Luhya in Webuye, Kenya; MXL, Mexican Ancestry in Los Angeles, California; PUR, Puerto Rican in Puerto Rico; TSI, Toscani in Italia; YRI, Yoruba in Ibadan, Nigeria.

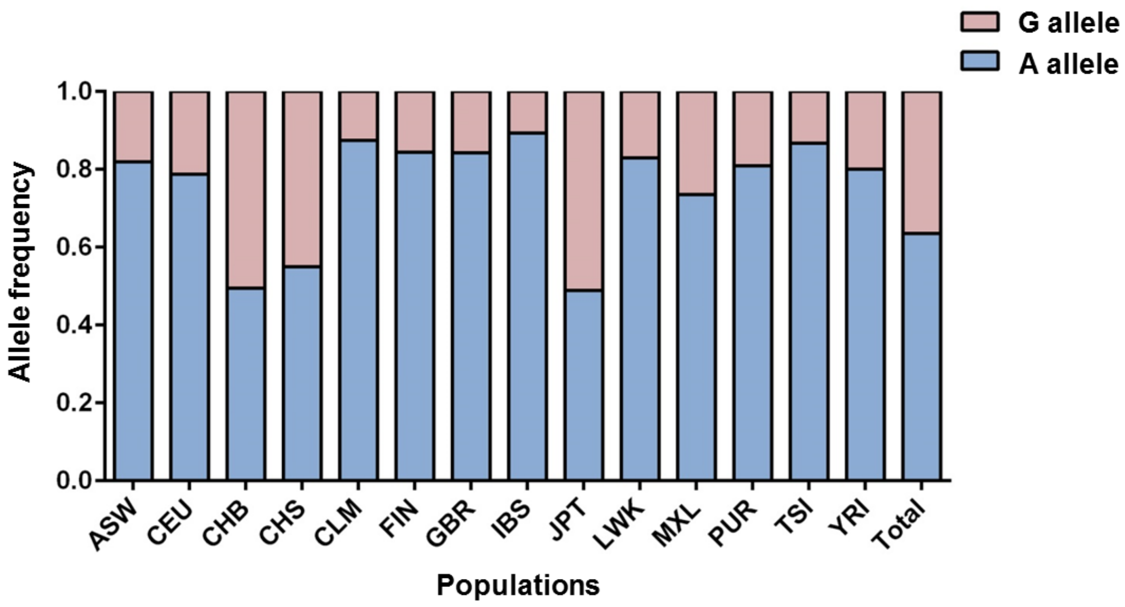

Supplementary Figure 17. Cluster plot distributions of rs2238126 in the three-stage GWAS study. The genotyping in the GWAS stage was conducted using Illumina HumanOmniZhongHua chips. The replication 1 and 2 stages were genotyped using the Sequenom and TaqMan methods, respectively.

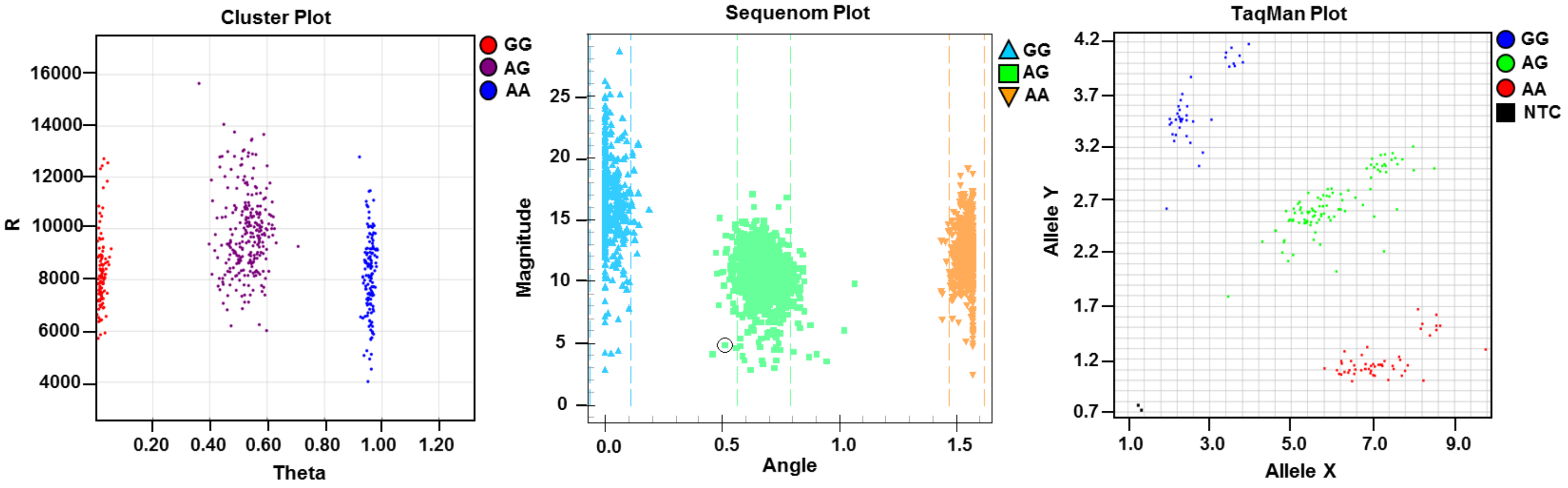

Supplementary Figure 18. Representative images of immunohistochemical staining for ETV6 in colorectal cancer tissues. (A) Negative staining, (B) weak staining, (C) moderate staining, and (D) strong staining. Magnification, × 40 (top) or × 200 (bottom).

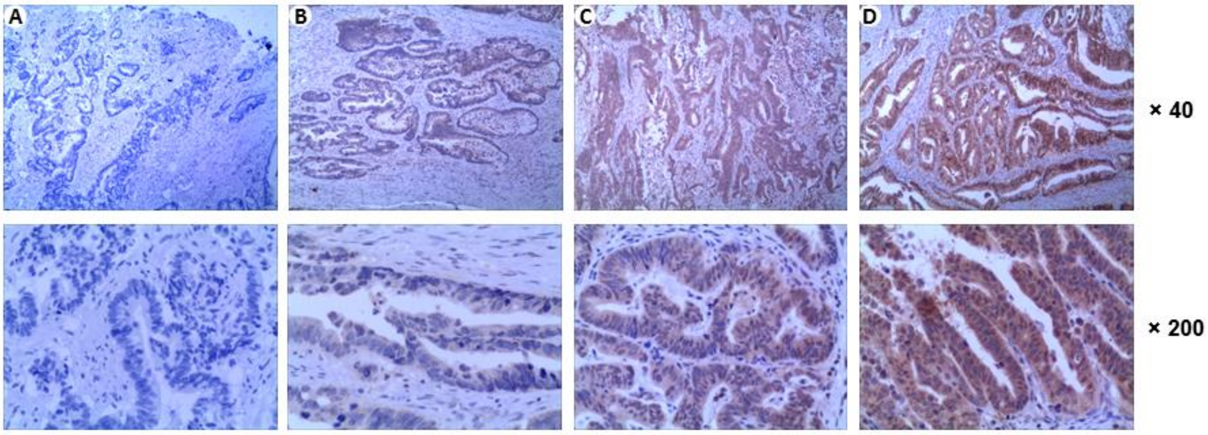

Supplementary Figure 19. Effect of *ETV6* overexpression and knockdown on colorectal cancer cell lines. (A) Overexpression of *ETV6* in SW480 cell line was confirmed by quantitative RT-PCR and Western blot. (B) Knockdown of *ETV6* in HT29 cell line was confirmed by quantitative RT-PCR and Western blot.

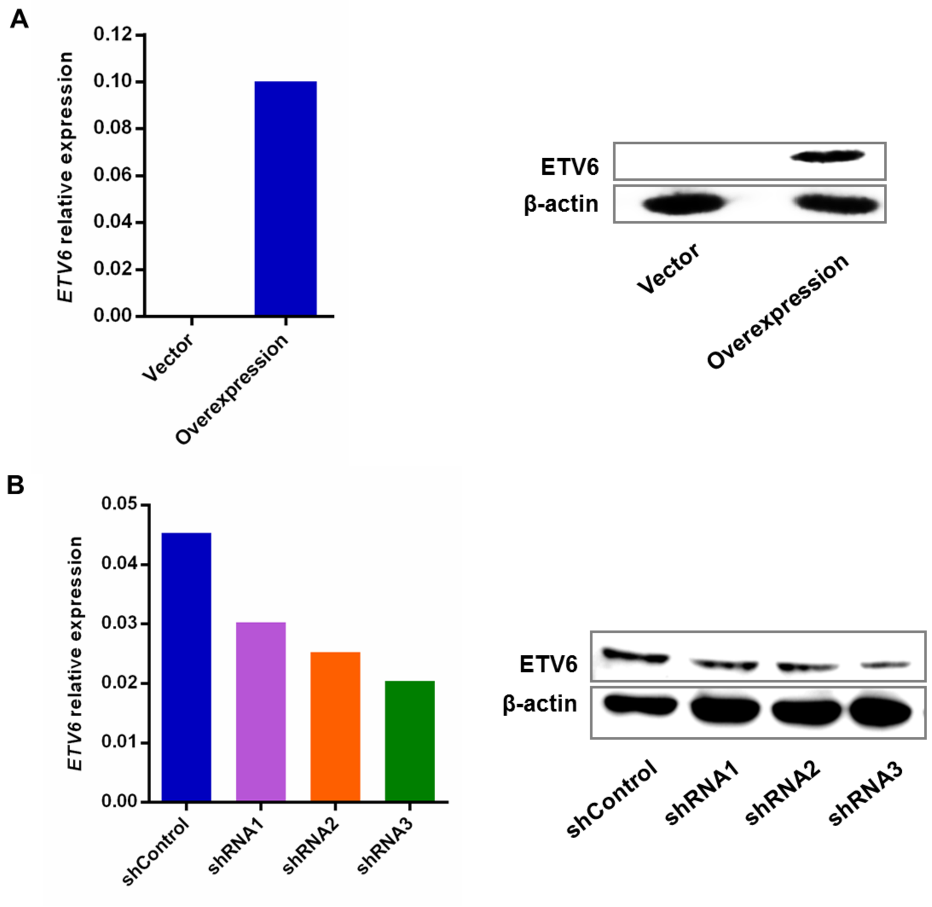

Supplementary Figure 20. Uncropped blots for Fig. 4A.

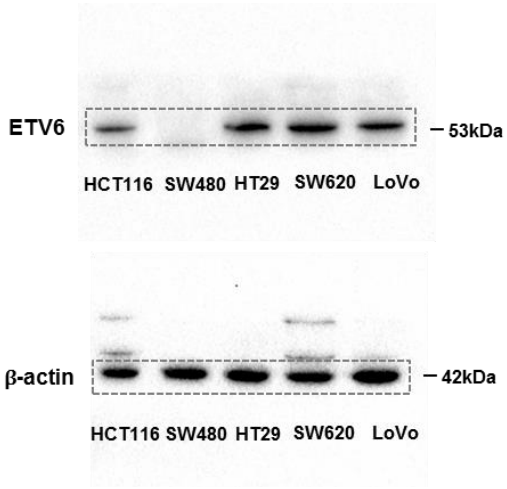

Supplementary Table 1. Characteristics of the subjects participated in this study

Study group	Population	Sample size		Age (year)		Sex, male (%)		Ever smokers (%)[a]		Tumor site (%)[a]	
		Cases	Controls	Mean ± SD	Range	Cases	Controls	Cases	Controls	Colon	Rectum
GWAS	Nanjing-1	1023	1306	62.5 ± 18.6	21-88	603 (58.9)	679 (52.0)	357 (34.9)	398 (30.5)	495 (48.4)	528 (51.6)
Replication 1	Nanjing-2	855	1258	59.8 ± 13.5	22-90	534 (62.5)	730 (58.0)	304 (35.6)	418 (33.2)	435 (51.4)	411 (48.6)
Replication 2											
Replication 2a	Wuhan	805	1200	59.2 ± 12.9	17-91	464 (57.6)	711 (59.3)	274 (34.0)	369 (30.8)	412 (53.2)	362 (46.8)
Replication 2b	Guangzhou	1179	1334	57.9 ± 12.1	18-89	728 (61.8)	881 (66.0)	643 (54.5)	566 (42.4)	304 (43.4)	397 (56.6)
Replication 2c	Nanjing-3	612	1188	60.1 ± 12.5	18-90	367 (60.0)	731 (61.5)	211 (34.5)	404 (34.0)	304 (49.7)	308 (50.3)
Replication 2d	Xi'an	643	384	60.2 ± 15.1	19-92	351 (54.6)	225 (58.6)	NA	NA	305 (47.6)	336 (52.4)
Replication 2e	Hangzhou	511	647	57.8 ± 14.5	19-98	306 (59.9)	376 (58.1)	151 (29.5)	187 (28.9)	256 (50.1)	255 (49.9)
Replication 2f	Shenyang	712	876	56.9 ± 14.3	18-89	409 (57.4)	471 (53.8)	NA	NA	NA	NA

[a] Some subjects were not available for the information. NA, not available.

Supplementary Table 2. Association results of previously reported SNPs in the GWAS stage

Chr	SNP	Position[a]	Nearby gene(s)	Previous GWAS reports			Reference	Our GWAS results		
				Allele[b]	RA[c]	OR (95% CI)		RAF (case/control)[d]	OR (95%CI)[e]	*P*[e]
1q25.3	rs10911251	183081194	*LAMC1*	A/C	A	1.09 (1.06-1.13)	12	0.567/0.535	1.14 (1.01-1.28)	3.11E-02
1q41	rs6691170	222045446	*DUSP10*	G/T	T	1.06 (1.03-1.09)	10	-	-	-
1q41	rs6687758	222164948	*DUSP10*	A/G	G	1.09 (1.06-1.12)	10	0.229/0.209	1.12 (0.98-1.29)	1.03E-01
2q32.3	rs11903757	192587204	*NABP1*	T/C	C	1.16 (1.10-1.22)	12	0.033/0.036	0.92 (0.67-1.27)	6.14E-01
3q26.2	rs10936599	169492101	*MYNN*	C/T	C	0.93 (0.91-0.96)	10	0.454/0.439	1.06 (0.95-1.19)	3.03E-01
5q31.1	rs647161	134499092	*PITX1*	C/A	A	1.17 (1.11-1.22)	17	0.253/0.268	0.92 (0.80-1.07)	2.94E-01
6p21.2	rs1321311	36622900	*CDKN1A*	G/T	T	1.10 (1.07-1.13)	11	0.155/0.173	0.87 (0.75-1.02)	9.63E-02
6q26-q27	rs7758229	160840252	*SLC22A3*	G/T	T	1.28 (1.18-1.39)	15	0.246/0.242	1.03 (0.90-1.17)	7.10E-01
8q23.3	rs16892766	117630683	*EIF3H*	A/C	C	1.25 (1.19-1.32)	7	-	-	-
8q24.21	rs10505477	128407443	-	G/A	A	1.17 (1.12-1.23)	4	0.459/0.420	1.18 (1.05-1.32)	6.65E-03
8q24.21	rs6983267	128413305	-	T/G	G	1.21 (1.15-1.27)	4,5	0.461/0.421	1.18 (1.05-1.32)	5.72E-03
8q24.21	rs7014346	128424792	-	G/A	A	1.19 (1.15-1.23)	4,8	0.340/0.307	1.17 (1.03-1.33)	1.36E-02
10p14	rs10795668	8701219	*FLJ3802842*	G/A	G	0.89 (0.86-0.91)	7	0.689/0.653	1.19 (1.05-1.35)	7.77E-03
10q22.3	rs704017	80819132	*ZMIZ1-AS1*	A/G	G	1.10 (1.06-1.13)	19	0.314/0.286	1.14 (1.01-1.30)	4.14E-02
10q25.2	rs11196172	114726843	*TCF7L2*	G/A	A	1.14 (1.10-1.18)	19	0.721/0.658	1.32 (1.17-1.50)	1.36E-05
10q26.12	rs1665650	118487100	*HSPA12A*	C/T	T	1.13 (1.08-1.19)	16	0.294/0.272	1.12 (0.97-1.28)	1.22E-01
11q12.2	rs174537	61552680	*MYRF*	G/T	G	1.16 (1.12-1.19)	19	0.683/0.733	0.82 (0.50-1.33)	4.19E-01
11q13.4	rs3824999	74345550	*POLD3*	A/C	C	1.08 (1.05-1.10)	11	0.403/0.382	1.09 (0.97-1.23)	1.56E-01
11q23	rs3802842	111171709	-	A/C	C	1.11 (1.08-1.15)	8	0.467/0.412	1.25 (1.11-1.41)	1.72E-04
12p13.32	rs10774214	4368352	*CCND2*	C/T	T	1.17 (1.11-1.23)	16	0.350/0.296	1.28 (1.11-1.48)	8.81E-04
12p13.32	rs3217810	4388271	*CCND2*	C/T	T	1.20 (1.12-1.28)	12	-	-	-
12p13.31	rs10849432	6385727	*CD9*	T/C	T	1.14 (1.09-1.18)	19	0.840/0.816	1.18 (1.01–1.38)	4.36E-02
12q13.13	rs7136702	50880216	*LARP4, DIP2B*	C/T	T	1.06 (1.04-1.08)	10	0.485/0.464	1.09 (0.93-1.27)	2.82E-01

12q13.13	rs11169552	51155663	*DIP2B, ATF1*	C/T	C	0.92 (0.90-0.95)	10	0.649/0.620	1.13 (1.01-1.28)	4.10E-02
12q24.21	rs59336	115116352	*TBX3*	T/A	T	1.09 (1.06-1.13)	12	0.730/0.751	0.90 (0.73-1.12)	3.53E-01
14q22.2	rs4444235	54410919	*BMP4*	T/C	C	1.11 (1.08-1.15)	9	0.463/0.469	0.98 (0.87-1.10)	7.03E-01
15q13.3	rs4779584	32994756	*SCG5, GREM1*	C/T	T	1.26 (1.19-1.34)	13	0.832/0.802	1.23 (1.06-1.43)	7.93E-03
16q22.1	rs9929218	68820946	*CDH1*	G/A	G	0.91 (0.89-0.94)	9	0.835/0.809	1.20 (1.03-1.40)	2.05E-02
17p13.3	rs12603526	800593	*NXN*	T/C	C	1.10 (1.06-1.14)	19	0.247/0.184	1.44 (1.24-1.68)	2.61E-06
18q21.1	rs7229639	46450976	*SMAD7*	G/A	A	1.22 (1.15-1.29)	18	0.206/0.170	1.26 (1.09-1.45)	2.20E-03
18q21.1	rs4939827	46453463	*SMAD7*	T/C	T	0.85 (0.81-0.89)	6,8	0.291/0.260	1.16 (1.02-1.32)	2.17E-02
19q13.1	rs10411210	33532300	*RHPN2*	C/T	C	0.87 (0.83-0.91)	9	0.846/0.817	1.23 (1.05-1.44)	8.81E-03
19q13.2	rs1800469	41860296	*TGFB1*	G/A	A	1.09 (1.06-1.12)	19	0.530/0.456	1.36 (1.21-1.53)	4.22E-07
20p12.3	rs961253	6404281	*BMP2*	C/A	A	1.12 (1.08-1.16)	9	0.097/0.086	1.14 (0.94-1.40)	1.87E-01
20p12.3	rs2423279	7812350	*HAO1*	T/C	C	1.14 (1.08-1.19)	17	0.379/0.324	1.28 (1.13-1.45)	9.88E-05
20q13.33	rs4925386	60921044	*LAMA5*	C/T	C	0.93 (0.91-0.95)	10	0.758/0.739	1.10 (0.96-1.26)	1.53E-01
Xp22.2	rs5934683	9751474	*SHROOM2*	C/T	T	1.07 (1.04-1.10)	11	-	-	-

[a] Based on the NCBI database, build 37.

[b] Major/minor allele.

[c] Risk allele derived from previous studies.

[d] Risk allele frequency.

[e] *P* value of additive model in the logistic regression analysis.

Supplementary Table 3. Association between the 53 selected SNPs and colorectal cancer risk in the GWAS stage

Chr	SNP	Position[a]	Gene	Location	Allele[b]	MAF (case/control)[c]	OR (95%CI)[d]	*P*[d]
1	rs1180275	40120085	*NT5C1A*	Intergenic	G/A	0.072/0.104	0.61 (0.48-0.77)	3.47E-05
1	rs7531902	95835124	*FLJ31662*	Intergenic	G/A	0.235/0.278	0.76 (0.66-0.89)	4.35E-04
1	rs4619033	204269242	*PLEKHA6*	Intron	A/G	0.128/0.158	0.70 (0.58-0.85)	2.69E-04
1	rs2180162	228866987	*RHOU*	Intron	A/G	0.244/0.200	1.41 (1.20-1.65)	3.12E-05
2	rs11681079	11080974	*KCNF1*	Intergenic	A/G	0.228/0.188	1.33 (1.13-1.57)	5.62E-04
2	rs4464317	69437291	*ANTXR1*	Intron	A/G	0.180/0.231	0.71 (0.61-0.84)	4.30E-05
2	rs16830810	135326853	*TMEM163*	Intron	A/G	0.131/0.169	0.69 (0.58-0.84)	1.09E-04
3	rs704417	64252424	*PRICKLE2*	Intergenic	A/G	0.100/0.142	0.61 (0.49-0.74)	1.91E-06
3	rs1488193	112681585	*CD200R1*	Intron	G/A	0.328/0.269	1.35 (1.16-1.56)	7.53E-05
3	rs10460813	112718063	*GTPBP8*	Intron	A/G	0.345/0.294	1.31 (1.13-1.51)	2.68E-04
3	rs12629188	172718955	*SPATA16*	Intron	A/G	0.297/0.350	0.79 (0.69-0.91)	9.41E-04
3	rs344944	187886274	*LPP*	Intron	A/G	0.117/0.081	1.67 (1.33-2.09)	1.19E-05
4	rs10021205	7151708	*FLJ36777*	Intergenic	A/G	0.227/0.276	0.74 (0.65-0.87)	9.37E-05
4	rs35699234	10654346	*CLNK*	Intron	A/G	0.278/0.324	0.76 (0.66-0.88)	2.34E-04
4	rs2375567	35796678	*ARAP2*	Intergenic	A/G	0.294/0.343	0.78 (0.68-0.91)	9.08E-04
4	rs13102452	126028567	*FAT4*	Intergenic	A/G	0.214/0.257	0.76 (0.65-0.90)	9.20E-04
5	rs418410	31687028	*C5orf22*	Intergenic	A/C	0.263/0.321	0.76 (0.66-0.88)	2.80E-04
5	rs6880261	167781775	*WWC1*	Intron	A/C	0.389/0.452	0.76 (0.67-0.87)	8.77E-05
5	rs157474	174948557	*SFXN1*	Intron	A/G	0.214/0.270	0.74 (0.64-0.87)	1.46E-04
6	rs2237143	15440339	*JARID2*	Intron	A/G	0.351/0.309	1.27 (1.11-1.46)	6.28E-04
6	rs3122160	55084697	*HCRTR2*	Intron	C/A	0.175/0.214	0.69 (0.58-0.81)	1.26E-05
6	rs1378720	105235975	*HACE1*	Intron	A/G	0.465/0.421	1.26 (1.11-1.44)	5.96E-04
6	rs1149321	105768250	*PREP*	Intron	G/A	0.297/0.243	1.28 (1.11-1.49)	9.32E-04
6	rs9383562	151582632	*AKAP12*	Intron	A/G	0.122/0.167	0.72 (0.59-0.87)	6.31E-04
7	rs11982650	2942260	*CARD11*	Intergenic	G/C	0.085/0.120	0.62 (0.50-0.77)	2.08E-05
7	rs6971374	9360629	*PER4*	Intergenic	C/A	0.098/0.132	0.65 (0.53-0.80)	4.11E-05
7	rs10251825	55603590	*VOPP1*	Intron	A/G	0.355/0.311	1.34 (1.17-1.54)	3.48E-05
7	rs2868895	77768217	*MAGI2*	Intron	A/G	0.334/0.278	1.29 (1.12-1.49)	4.33E-04
7	rs17165493	88556361	*ZNF804B*	Intron	G/A	0.163/0.208	0.73 (0.61-0.87)	3.28E-04
7	rs10954366	131855368	*PLXNA4*	Intron	G/A	0.153/0.185	0.74 (0.62-0.88)	7.74E-04
8	rs56910844	40246578	*C8orf4*	Intergenic	A/G	0.391/0.321	1.33 (1.16-1.53)	6.88E-05
9	rs2109664	122301156	*DBC1*	Intergenic	G/A	0.470/0.422	1.28 (1.12-1.47)	3.76E-04
10	rs210280	43089965	*ZNF33B*	Missense	A/G	0.143/0.114	1.43 (1.17-1.74)	5.73E-04
10	rs2804018	134617102	*NKX6-2*	Intergenic	A/G	0.452/0.384	1.27 (1.11-1.45)	5.03E-04
11	rs1374494	35593571	*FJX1*	Intergenic	A/G	0.088/0.131	0.65 (0.52-0.81)	9.43E-05
11	rs688099	121072088	*TECTA*	Intergenic	C/A	0.081/0.117	0.66 (0.53-0.82)	1.51E-04
12	rs2238126	12009741	*ETV6*	Intron	A/G	0.526/0.474	1.25 (1.10-1.43)	7.41E-04
12	rs2363074	94224637	*CRADD*	Intron	G/A	0.233/0.280	0.74 (0.64-0.86)	7.92E-05

14	rs7157453	55159204	*SAMD4A*	Intron	A/G	0.107/0.144	0.63 (0.52-0.78)	9.15E-06
14	rs1742083	91186670	*TTC7B*	Intron	G/A	0.395/0.464	0.76 (0.67-0.87)	6.35E-05
15	rs1668543	42340820	*PLA2G4E*	Intron	G/A	0.147/0.115	1.43 (1.17-1.75)	4.28E-04
15	rs1370276	80450328	*FAH*	Intron	G/A	0.116/0.150	0.70 (0.58-0.86)	4.38E-04
16	rs16959059	69733009	*NFAT5*	3'-UTR	G/A	0.409/0.355	1.26 (1.10-1.45)	9.18E-04
16	rs4247109	69803443	*WWP2*	Intron	A/G	0.437/0.380	1.27 (1.11-1.45)	5.36E-04
17	rs10521202	12814564	*ARHGAP44*	Intron	A/G	0.471/0.422	1.26 (1.10-1.43)	7.54E-04
17	rs2948541	25860210	*KSR1*	Intron	C/A	0.270/0.325	0.77 (0.66-0.89)	4.75E-04
18	rs4517886	20562885	*RBBP8*	Intron	C/A	0.420/0.373	1.32 (1.15-1.51)	7.22E-05
18	rs57786382	39463007	*PIK3C3*	Intergenic	G/A	0.052/0.088	0.57 (0.44-0.74)	2.47E-05
19	rs3745765	37854235	*HKR1*	Missense	C/A	0.333/0.290	1.28 (1.11-1.47)	7.93E-04
19	rs4452075	37879589	*ZNF527*	Missense	A/G	0.326/0.278	1.34 (1.16-1.55)	8.25E-05
19	rs1015849	37946180	*ZNF569*	Intron	G/A	0.256/0.210	1.35 (1.16-1.58)	1.80E-04
21	rs9978525	34947856	*SON*	Intron	A/C	0.480/0.422	1.25 (1.10-1.43)	9.12E-04
22	rs929271	30638226	*LIF*	Intron	A/C	0.331/0.385	0.74 (0.65-0.85)	2.17E-05

[a] Based on the NCBI database, build 37.

[b] Major/minor allele.

[c] Minor allele frequency.

[d] *P* value of additive model with adjustment for top eigen, age and sex.

Supplementary Table 4. Association of the 53 SNPs with colorectal cancer risk in the replication 1 stage

Chr	SNP	Position[a]	Gene	Location	Allele[b]	MAF (case/control)[c]	OR (95%CI)[d]	*P*[d]
1	rs1180275	40120085	*NT5C1A*	Intergenic	G/A	0.077/0.082	0.91 (0.72-1.15)	4.41E-01
1	rs7531902	95835124	*FLJ31662*	Intergenic	G/A	0.255/0.254	1.01 (0.88-1.16)	8.99E-01
1	rs4619033	204269242	*PLEKHA6*	Intron	A/G	0.157/0.161	0.98 (0.82-1.16)	7.87E-01
1	rs2180162	228866987	*RHOU*	Intron	A/G	0.214/0.221	0.97 (0.83-1.12)	6.44E-01
2	rs11681079	11080974	*KCNF1*	Intergenic	A/G	0.217/0.211	1.02 (0.88-1.18)	7.93E-01
2	rs4464317	69437291	*ANTXR1*	Intron	A/G	0.207/0.198	1.06 (0.91-1.23)	4.93E-01
2	rs16830810	135326853	*TMEM163*	Intron	A/G	0.130/0.133	0.97 (0.81-1.17)	7.79E-01
3	rs704417	64252424	*PRICKLE2*	Intergenic	A/G	0.113/0.126	0.89 (0.74-1.08)	2.27E-01
3	rs1488193	112681585	*CD200R1*	Intron	G/A	0.293/0.305	0.94 (0.82-1.08)	3.89E-01
3	rs10460813	112718063	*GTPBP8*	Intron	A/G	0.306/0.315	0.96 (0.84-1.1)	5.69E-01
3	rs12629188	172718955	*SPATA16*	Intron	A/G	0.340/0.357	0.93 (0.82-1.06)	2.67E-01
3	rs344944	187886274	*LPP*	Intron	A/G	0.103/0.104	0.99 (0.81-1.21)	9.46E-01
4	rs10021205	7151708	*FLJ36777*	Intergenic	A/G	0.214/0.229	0.95 (0.82-1.13)	5.33E-01
4	rs35699234	10654346	*CLNK*	Intron	A/G	0.317/0.302	1.08 (0.94-1.23)	2.80E-01
4	rs2375567	35796678	*ARAP2*	Intergenic	A/G	0.330/0.327	1.02 (0.89-1.16)	7.70E-01
4	rs13102452	126028567	*FAT4*	Intergenic	A/G	0.226/0.248	0.88 (0.76-1.02)	8.66E-02
5	rs418410	31687028	*C5orf22*	Intergenic	A/C	0.348/0.305	1.21 (1.07-1.38)	3.45E-03
5	rs6880261	167781775	*WWC1*	Intron	A/C	0.403/0.409	0.98 (0.86-1.11)	7.10E-01
5	rs157474	174948557	*SFXN1*	Intron	A/G	0.241/0.235	1.03 (0.89-1.2)	6.64E-01
6	rs2237143	15440339	*JARID2*	Intron	A/G	0.339/0.330	1.05 (0.92-1.19)	4.99E-01
6	rs3122160	55084697	*HCRTR2*	Intron	C/A	0.226/0.196	1.18 (1.02-1.37)	2.70E-02
6	rs1378720	105235975	*HACE1*	Intron	A/G	0.443/0.447	0.98 (0.86-1.11)	7.22E-01
6	rs1149321	105768250	*PREP*	Intron	G/A	0.266/0.259	1.04 (0.9-1.19)	5.87E-01
6	rs9383562	151582632	*AKAP12*	Intron	A/G	0.151/0.154	0.97 (0.81-1.15)	7.13E-01
7	rs11982650	2942260	*CARD11*	Intergenic	G/C	0.107/0.118	0.9 (0.74-1.1)	3.05E-01
7	rs6971374	9360629	*PER4*	Intergenic	C/A	0.121/0.125	0.96 (0.8-1.16)	6.60E-01
7	rs10251825	55603590	*VOPP1*	Intron	A/G	0.321/0.334	0.94 (0.83-1.08)	3.84E-01
7	rs2868895	77768217	*MAGI2*	Intron	A/G	0.303/0.306	0.99 (0.86-1.13)	8.41E-01
7	rs17165493	88556361	*ZNF804B*	Intron	G/A	0.183/0.201	0.89 (0.76-1.04)	1.49E-01
7	rs10954366	131855368	*PLXNA4*	Intron	G/A	0.173/0.166	1.04 (0.88-1.23)	6.25E-01
8	rs56910844	40246578	*C8orf4*	Intergenic	A/G	0.366/0.368	0.99 (0.88-1.13)	9.35E-01
9	rs2109664	122301156	*DBC1*	Intergenic	G/A	0.429/0.430	1 (0.88-1.13)	9.75E-01
10	rs210280	43089965	*ZNF33B*	Missense	A/G	0.106/0.118	0.89 (0.72-1.08)	2.34E-01
10	rs2804018	134617102	*NKX6-2*	Intergenic	A/G	0.413/0.420	0.98 (0.86-1.11)	7.10E-01
11	rs1374494	35593571	*FJX1*	Intergenic	A/G	0.109/0.101	1.09 (0.89-1.33)	3.94E-01
11	rs688099	121072088	*TECTA*	Intergenic	C/A	0.101/0.104	0.97 (0.82-1.20)	8.75E-01
12	rs2238126	12009741	*ETV6*	Intron	A/G	0.523/0.478	1.20 (1.06-1.36)	4.46E-03
12	rs2363074	94224637	*CRADD*	Intron	G/A	0.258/0.243	1.1 (0.95-1.26)	2.09E-01

14	rs7157453	55159204	*SAMD4A*	Intron	A/G	0.128/0.134	0.95 (0.8-1.15)	6.18E-01
14	rs1742083	91186670	*TTC7B*	Intron	G/A	0.427/0.449	0.93 (0.82-1.05)	2.14E-01
15	rs1668543	42340820	*PLA2G4E*	Intron	G/A	0.133/0.139	0.95 (0.79-1.14)	5.69E-01
15	rs1370276	80450328	*FAH*	Intron	G/A	0.142/0.126	1.15 (0.96-1.38)	1.34E-01
16	rs16959059	69733009	*NFAT5*	3'-UTR	G/A	0.381/0.370	1.06 (0.91-1.15)	3.78E-01
16	rs4247109	69803443	*WWP2*	Intron	A/G	0.413/0.401	1.05 (0.92-1.19)	4.90E-01
17	rs10521202	12814564	*ARHGAP44*	Intron	A/G	0.426/0.432	0.98 (0.86-1.11)	7.06E-01
17	rs2948541	25860210	*KSR1*	Intron	C/A	0.303/0.302	1 (0.87-1.14)	9.50E-01
18	rs4517886	20562885	*RBBP8*	Intron	C/A	0.379/0.373	1.03 (0.9-1.17)	6.88E-01
18	rs57786382	39463007	*PIK3C3*	Intergenic	G/A	0.065/0.073	0.86 (0.68-1.1)	2.21E-01
19	rs3745765	37854235	*HKR1*	Missense	C/A	0.318/0.315	1.01 (0.89-1.15)	8.57E-01
19	rs4452075	37879589	*ZNF527*	Missense	A/G	0.295/0.302	0.97 (0.85-1.1)	6.10E-01
19	rs1015849	37946180	*ZNF569*	Intron	G/A	0.236/0.240	0.98 (0.85-1.13)	7.84E-01
21	rs9978525	34947856	*SON*	Intron	A/C	0.475/0.449	1.11 (0.98-1.26)	9.84E-02
22	rs929271	30638226	*LIF*	Intron	A/C	NA	NA	NA

[a] Based on the NCBI database, build 37.

[b] Major/minor allele.

[c] Minor allele frequency.

[d] *P* value of additive model with adjustment for age and sex.

Supplementary Table 5. LD ($r^2 > 0.10$) and association between rs2238126 and SNPs identified by genotyping or imputation analysis at chromosome 12p13.2

Chr	SNP	Position[a]	Allele[b]	Status	*P*[c]	LD[d]
12	rs2515767	11977949	T/C	Genotyped	3.36E-02	0.109
12	rs2855736	11981298	G/A	Genotyped	3.08E-02	0.108
12	rs7138674	12004154	C/T	Genotyped	1.92E-02	0.631
12	rs2238122	12004731	C/T	Genotyped	1.98E-02	0.107
12	rs2238123	12005032	G/A	Imputed	1.37E-02	0.198
12	rs1894307	12005720	C/T	Genotyped	1.98E-03	0.658
12	rs12319153	12006800	T/G	Imputed	7.10E-03	0.104
12	rs743613	12008864	G/A	Genotyped	1.41E-02	0.300
12	rs10772508	12009529	A/G	Genotyped	1.82E-02	0.305
12	rs2238126	12009741	A/G	Genotyped	7.41E-04	1.000
12	rs743614	12009874	G/A	Imputed	1.78E-02	0.303
12	rs10772509	12010056	G/A	Imputed	2.77E-02	0.303
12	rs4486711	12010544	G/T	Imputed	1.61E-02	0.307
12	rs2255953	12010736	C/T	Genotyped	2.01E-03	0.655
12	rs2238128	12012580	C/T	Imputed	7.15E-03	0.104
12	rs2723803	12013189	A/G	Imputed	1.05E-02	0.200
12	rs2855707	12013292	G/A	Imputed	9.74E-03	0.201
12	rs2855708	12013572	G/A	Genotyped	9.10E-04	0.660
12	rs6488463	12013612	A/G	Imputed	2.18E-02	0.198
12	rs2855709	12013985	C/A	Genotyped	3.41E-02	0.190
12	rs2238129	12014424	G/A	Imputed	2.80E-02	0.191
12	rs2283339	12015706	G/T	Genotyped	7.95E-03	0.207
12	rs2238130	12016008	A/G	Genotyped	3.19E-02	0.118
12	rs2416944	12016045	C/T	Genotyped	6.57E-03	0.163
12	rs928936	12016183	G/T	Genotyped	5.17E-03	0.160
12	rs12305013	12019067	A/G	Genotyped	3.26E-03	0.108
12	rs11054476	12019372	T/C	Imputed	2.04E-03	0.109
12	rs12305522	12019939	A/G	Genotyped	7.20E-03	0.108
12	rs2723805	12020114	C/T	Imputed	1.39E-02	0.248
12	rs7973930	12020170	C/T	Genotyped	7.20E-03	0.107
12	rs11054477	12020451	A/C	Imputed	3.45E-02	0.107
12	rs10772510	12021657	T/C	Genotyped	3.11E-02	0.103
12	rs2239171	12023773	C/A	Genotyped	9.76E-03	0.239
12	rs2239172	12023941	C/T	Genotyped	1.24E-02	0.236
12	rs4763731	12033871	C/T	Genotyped	6.45E-03	0.126
12	rs11054481	12035282	C/G	Genotyped	3.45E-02	0.106
12	rs2710310	12035649	C/T	Genotyped	1.90E-02	0.108

[a] Based on the NCBI database, build 37.

[b] Major/minor allele.

[c] *P* value of additive model with adjustment for age and sex.

[d] LD value (r^2) of the index SNP rs2238126 with other SNPs at 12p13.2.

Supplementary Table 6. Subgroup analyses of the association between rs2238126 and colorectal cancer risk

Variables	MAF[a]		OR (95%CI)[b]	*P*[c]	P_{het}[d]
	**Cases**	**Controls**			
Age, years*					
≤60	0.528	0.471	1.17 (1.09-1.25)	8.11×10^{-6}	0.729
>60	0.522	0.482	1.19 (1.11-1.27)	1.05×10^{-6}	
Sex					
Male	0.519	0.473	1.20 (1.13-1.28)	8.68×10^{-9}	0.318
Female	0.511	0.481	1.14 (1.05-1.23)	8.67×10^{-4}	
Smoking status					
Never	0.524	0.479	1.21 (1.12-1.30)	3.13×10^{-7}	0.537
Ever	0.512	0.473	1.17 (1.09-1.27)	4.54×10^{-5}	
Tumor site					
Colon	0.521	0.476	1.18 (1.11-1.26)	1.42×10^{-7}	0.567
Rectum	0.512	0.476	1.15 (1.08-1.22)	1.41×10^{-5}	

[a] The age cutoff at 60 years was used based on the median age of controls.

[b] Minor allele frequency of G allele.

[c] *P* value of additive model with adjustment for age and sex where is appropriate.

[d] *P* value for the heterogeneity.

Supplementary Table 7. Association between the rs2238126 G allele and age at diagnosis of colorectal cancer

**Population**	**N[a]**	**Effect (years)**	**SE**	***P***	**MAF[b]**	**P_{het}[c]**	**I^2**
Nanjing-1	1023	-0.478	0.544	0.380	0.526		
Nanjing-2	855	-1.269	0.574	0.027	0.523		
Wuhan	805	-1.442	0.626	0.021	0.504		
Guangzhou	1179	-0.693	0.608	0.254	0.517		
Nanjing-3	612	-1.771	0.748	0.018	0.507		
Xi'an	643	-1.118	0.738	0.130	0.508		
Hangzhou	511	-1.664	0.771	0.031	0.526		
Shenyang	712	-0.642	0.434	0.139	0.504		
Combined[d]	6340	-1.007	0.212	1.98×10^{-6}	0.515	0.732	0

[a] Colorectal cancer cases in each group.

[b] Minor allele frequency of G allele.

[c] *P* value for the heterogeneity.

[d] Combined by meta-analysis under a fixed-effects model.

Supplementary Table 8. Cumulative effect of rs2238126 and previously associated SNPs on the risk of colorectal cancer in the GWAS stage

**Counts of risk alleles**[a]	**Cases**[b]		**Controls**[b]		**OR (95% CI)**[c]	P^c	P_{trend}
	**N**	**%**	**N**	**%**			
<15	38	3.7	119	9.1	1.00		
15-17	139	13.6	306	23.4	1.41 (0.93-2.14)	0.104	
18-20	333	32.6	454	34.8	2.28 (1.54-3.38)	3.76×10^{-5}	
21-23	322	31.4	310	23.7	3.25 (2.18-4.84)	6.22×10^{-9}	
≥24	191	18.7	117	9.0	5.09 (3.30-7.84)	1.60×10^{-13}	2.34×10^{-24}

[a] Based on rs2238126, rs10911251, rs10505477, rs6983267, rs7014346, rs10795668, rs704017, rs11196172, rs3802842, rs10774214, rs10849432, rs11169552, rs4779584, rs9929218, rs12603526, rs7229639, rs4939827, rs10411210, rs1800469, rs2423279. These SNPs were significantly associated with colorectal cancer risk in the GWAS stage.

[b] All the cases and controls were from the GWAS stage.

[c] Logistic regression analysis with ajustment for age and sex.

Supplementary Table 9. Genotype and allele distributions of rs2238126 in samples from the 1000 Genomes Project

Populations	Sample size	Genotype, n (%)			Allele, n (%)	
		**AA**	**AG**	**GG**	**A**	**G**
Our study[a]						
GWAS	1306	373 (0.285)	629 (0.482)	304 (0.233)	1375 (0.526)	1237 (0.474)
Replication 1	1254	347 (0.277)	615 (0.490)	292 (0.233)	1309 (0.522)	1199 (0.478)
Replication 2	5629	1489 (0.264)	2908 (0.517)	1232 (0.219)	5886 (0.523)	5372 (0.477)
Total						
The 1000 Genomes Project[b]						
ASW	61	41 (0.672)	18 (0.295)	2 (0.033)	100 (0.820)	22 (0.180)
CEU	85	53 (0.624)	28 (0.329)	4 (0.047)	134 (0.788)	36 (0.212)
CHB	97	25 (0.258)	46 (0.474)	26 (0.268)	96 (0.495)	98 (0.505)
CHS	100	32 (0.320)	46 (0.460)	22 (0.220)	110 (0.550)	90 (0.450)
CLM	60	46 (0.767)	13 (0.217)	1 (0.016)	105 (0.875)	15 (0.125)
FIN	93	68 (0.731)	21 (0.226)	4 (0.043)	157 (0.844)	29 (0.156)
GBR	89	62 (0.697)	26 (0.292)	1 (0.011)	150 (0.843)	28 (0.157)
IBS	14	11 (0.786)	3 (0.214)	0 (0.000)	25 (0.893)	3 (0.107)
JPT	89	23 (0.258)	41 (0.461)	25 (0.281)	87 (0.489)	91 (0.511)
LWK	97	65 (0.670)	31 (0.320)	1 (0.010)	161 (0.830)	33 (0.170)
MXL	66	38 (0.576)	21 (0.318)	7 (0.106)	97 (0.735)	35 (0.265)
PUR	55	36 (0.655)	17 (0.309)	2 (0.036)	89 (0.809)	21 (0.191)
TSI	98	73 (0.745)	24 (0.245)	1 (0.010)	170 (0.867)	26 (0.133)
YRI	88	55 (0.625)	31 (0.352)	2 (0.023)	141 (0.801)	35 (0.199)
Total	1092	487 (0.446)	413 (0.378)	192 (0.176)	1387 (0.635)	797 (0.365)

[a] Only in the controls.

[b] ASW, African Ancestry in Southwest USA; CEU, (CEPH) with Northern and Western European ancestry; CHB , Han Chinese in Beijing, China; CHS, Han Chinese South; CLM, Colombian in Medellin, Colombia; FIN, Finnish in Finland; GBR, British in England and Scotland ; IBS, Iberian populations in Spain; JPT, Japanese in Tokyo, Japan; LWK, Luhya in Webuye, Kenya; MXL, Mexican Ancestry in Los Angeles, California; PUR, Puerto Rican in Puerto Rico; TSI, Toscani in Italia; YRI, Yoruba in Ibadan, Nigeria.

Supplementary Table 10. Sequences of primers and probes used in this study

Experiment	Description	Sequence (5'-3')		
Seqenom	SNP_ID	1st-PCRP	2nd-PCRP	EXT1_SEQ
Genotyping	rs57786382	ACGTTGGATGCCACCCCACACTTAATATTC	ACGTTGGATGCTCAATTCATTGGACTCCTG	GACTCCTGCTTCATCTTC
Genotyping	rs17165493	ACGTTGGATGGCAGCGCATACATTTTGGG	ACGTTGGATGCAAGGCACACTTGGGAATAC	CAACACCATGAAGGGCAC
Genotyping	rs56910844	ACGTTGGATGATGCAATGGAGGCATTGGTC	ACGTTGGATGAGGCAGCTGTCAGTCTCTTC	CTGTTCTCCCCTGACTTAC
Genotyping	rs12629188	ACGTTGGATGTAGAACAATTCCCCTACCCC	ACGTTGGATGAGTGGGTAAAAGGAACTGC	AGACTACGACGAGTAATGC
Genotyping	rs2180162	ACGTTGGATGCCAAGTCCAGAAATGTTCCC	ACGTTGGATGATAAGGGCTGTGATAGGGAC	aTGATAGGGACAAGCACAA
Genotyping	rs9978525	ACGTTGGATGTGAGGTTCAGACTCCTACAG	ACGTTGGATGTCTCCTACTGCAACAAGACC	AAATTTCAAAGCACCACTTG
Genotyping	rs1742083	ACGTTGGATGGTCAGAGAGAAATCAGATCG	ACGTTGGATGGAAATGGGTACCTTTCCTCC	ccCCTTTCCTCCTCAATGCTA
Genotyping	rs4517886	ACGTTGGATGTTAACTCACTCAAGGTCAGC	ACGTTGGATGTGGCAAGACCTGGAGAGTAG	GAGAGTAGGAAAGTTCAGAG
Genotyping	rs1374494	ACGTTGGATGAGGAACCAAATGCCTTTAGC	ACGTTGGATGGTGATAAAACACTTGAAGGG	AAGGGGTCCATAATACATCAC
Genotyping	rs2109664	ACGTTGGATGGGAATTTGGCAAAGAAGGAG	ACGTTGGATGCTGTCCATCATAAAGATTTG	gTCATGTGACAATTCCAGAGA
Genotyping	rs4619033	ACGTTGGATGGGTTATCTATGCCATTGCCC	ACGTTGGATGTCTGCTGAAGATGCACCCTG	CACCCTGCTGTTAACACATCAC
Genotyping	rs1180275	ACGTTGGATGGCCTCTTAGGCAATGATCAG	ACGTTGGATGCAACACTGGGCACTTCAATC	aGCACTTCAATCTATCCAAAAC
Genotyping	rs2375567	ACGTTGGATGGCAGTGTTTATGTCCCCTAC	ACGTTGGATGGCATCTAACATTGGACAACC	CGATCTAAAGGGAAAGATCAGC
Genotyping	rs7157453	ACGTTGGATGTAGGTTACGTGGACAATGCC	ACGTTGGATGCTGTTGCACAAGAGGCTCTA	ggacGAGGCTCTAGACATGAGA
Genotyping	rs1370276	ACGTTGGATGTGGGCTTGGCTCATTTACTC	ACGTTGGATGAACATGGTTCTTCAGTCCGC	tcaCTGTGGACTCTTCAATAGAC
Genotyping	rs418410	ACGTTGGATGGCAGGTGAGCAGAGATTCC	ACGTTGGATGCTAGGCTTTTCATACCTCTTT	agTTGGAGGGTTCTGGAACAAGG
Genotyping	rs16830810	ACGTTGGATGGAAAAACCCTAAGGGTGGTG	ACGTTGGATGCTCATAACCAGGTATCAGGG	ctgccGTGAACTGAACACTGCCAC
Genotyping	rs35699234	ACGTTGGATGGCAAATAACTATCAGGAAAC	ACGTTGGATGAAGACCAGGCATTAAAAAC	AGGCATTAAAAACATTTAAAGTAA
Genotyping	rs3122160	ACGTTGGATGGGTTATCCAATTTGTTAGTG	ACGTTGGATGGCAATCAAAGAGGCAGGAAC	cGAGGCAGGAACAATAAAGATTTG
Genotyping	rs2868895	ACGTTGGATGCTTGCCTATGGCTTCAAACC	ACGTTGGATGAGTCATTCGCTTGCTCTTCG	TTCGTAATTCAAATTGCTTTCAACC
Genotyping	rs10251825	ACGTTGGATGTCAGGTGTAGGAAAGAGTCG	ACGTTGGATGGGGCAGGCAGACATCAACTT	acaaGCAGACATCAACTTACCGCAA
Genotyping	rs1149321	ACGTTGGATGGTGAGGGATCTGGTGTCATA	ACGTTGGATGTACCCACAGCTATAATCAGG	aAATCAGGAATGGATGAAGGCTGTA
Genotyping	rs1015849	ACGTTGGATGGCTTAAAGTTGAGTTCCTCC	ACGTTGGATGGTAGCAATTGGTAAGGCCTC	ccctcGGTAAGGCCTCAGAAAAGACA
Genotyping	rs13102452	ACGTTGGATGGGAAAGAAAGAAGTAAAACCC	ACGTTGGATGAGTAGCTTACATGTGGTTTC	tctgTGGGATTTTTTAGTACACAGAA

Genotyping	rs4452075	ACGTTGGATGTCTTCCCCCAAAATTCAGTC	ACGTTGGATGACTCTGGTTGAATTCTTCAC	ccCCTATCAAAGATTCCTTCTCAGCAC
Genotyping	rs3745765	ACGTTGGATGTCGTCCACACTCAGCACATA	ACGTTGGATGGAGGCTTTACCCGGAAATC	caGCTTTACCCGGAAATCAACCCTGAG
Genotyping	rs704417	ACGTTGGATGACTCAACTAGCTGCACAGAT	ACGTTGGATGGCAGTTGTTTAGGTGGTGTG	TTCCCTGCTTTCTGTACA
Genotyping	rs11982650	ACGTTGGATGGGATTACAGGATTTCCTCTC	ACGTTGGATGGTGACCTGGTTAGGTAGAAG	CGGGCCGACACTACCAGC
Genotyping	rs6971374	ACGTTGGATGAAGGAGTACAGCAGCAAGCG	ACGTTGGATGTTCTGCTCGAATTAGGCCAC	TAGGCCACCTAGGGATTG
Genotyping	rs2948541	ACGTTGGATGTCCTGGGTTCAGTTTCTTGC	ACGTTGGATGAGAGGCGTAAACTGAGGATG	tAGGATGAGGAACAGTCCC
Genotyping	rs6880261	ACGTTGGATGGGAAGCTCAATGCTCTCTAC	ACGTTGGATGTTCTCAGGGCCCGTTCTTTG	TTCTTTGGTCATTTCCAGTG
Genotyping	rs9383562	ACGTTGGATGGTATTTTTTAGAGACGGGTTG	ACGTTGGATGAGAGGCGGGTGGATCAACT	ggCGGGAGTTCGAGACCACA
Genotyping	rs4464317	ACGTTGGATGGGCAGAGTGTTTTTGGATGG	ACGTTGGATGTGGCTGATGGATGGCTTATC	TGGCTTATCCCTCTTTACACC
Genotyping	rs1668543	ACGTTGGATGATGAAGTTGGAGCAATGCGG	ACGTTGGATGTGCACTGTGACAGGCATGG	ccGCGGAGGGGAGAAATCACA
Genotyping	rs344944	ACGTTGGATGAAGCATTAGAGAGGTCTAGG	ACGTTGGATGAAAACATGGTCACCTGGTTC	ccccCACCTGGTTCTTCAGTTA
Genotyping	rs11681079	ACGTTGGATGGGCAAACTGGAGCATATGAC	ACGTTGGATGTCCAAGATGAGCTCTGCAAG	CCATCATCTGTAACTTGTAGCC
Genotyping	rs2804018	ACGTTGGATGACAATTCATTGGGACCGGAG	ACGTTGGATGGCTCTTCATAATTGTGTGGC	cctcATTGTGTGGCCATTAGCC
Genotyping	rs929271	ACGTTGGATGAAGAACAGTGTGAACCAGCC	ACGTTGGATGCTGGGCCAATTTGTGGAGAG	ggaatGGTGCCTTTCTGTCTTGC
Genotyping	rs10954366	ACGTTGGATGCTGGATCTGACTGAACCCG	ACGTTGGATGGCAGAACATTCATGATCCTC	agggtTTCCAGGCTCAGGGCAAA
Genotyping	rs210280	ACGTTGGATGCCATTTAACATGGACGTAAG	ACGTTGGATGCTGAAACAGTGTTGAAACTC	cccatTGTTGAAACTCATTCCACA
Genotyping	rs1488193	ACGTTGGATGTCTCTAACATCAGAGACTGC	ACGTTGGATGTCAGCTAAAGTGTCTGCAAG	cttCAAGCCAGATATAACCCATGA
Genotyping	rs2237143	ACGTTGGATGCCTTCTTGGAAACGTTTTGAC	ACGTTGGATGCGTACGGCTTAAATGAACCC	gggtAACCCTCAAAGGGTGGGACA
Genotyping	rs7531902	ACGTTGGATGCTTTTGAACAGAAAAACCTGC	ACGTTGGATGCCAGATAATCCATCTCCAATG	CCATCTCCAATGAGTATATATGACA
Genotyping	rs2238126	ACGTTGGATGGTGGGCATATGTTAACATAC	ACGTTGGATGCACTGGGGAAGTAATATTTG	gtgGATTTTTTTTTCAGAACCAGAC
Genotyping	rs2363074	ACGTTGGATGAGGTCGTGTTGAAGCCTAAG	ACGTTGGATGCTAACGATACAGAAATGGGC	TAGCTATTTCTGTAAATTGTTTGGA
Genotyping	rs157474	ACGTTGGATGACAAGGAAACGGAAGCTTGG	ACGTTGGATGTGGTGCCAAGGTCTCAAGTC	ctAAGGTCTCAAGTCTCCCGCCTTCC
Genotyping	rs10460813	ACGTTGGATGCTCCAGGACCCTTCTCATTG	ACGTTGGATGAGTATACACTGTATGTCTGC	cccccCTGTATGTCTGCTGCTTAATA
Genotyping	rs4247109	ACGTTGGATGTTTTCCCTGGCTCCTTCTAC	ACGTTGGATGCAGCTCTGAGTCAAGAAACC	gggtAGTCAAGAAACCAGTGAACACC
Genotyping	rs1378720	ACGTTGGATGCAGATATAGTATGCAATGG	ACGTTGGATGGAAAATAACTTTAATTTCTAAC	AAATAACTTTAATTTCTAACTATGGTA
Genotyping	rs10521202	ACGTTGGATGCAGATGCATGTTCAATCTGTC	ACGTTGGATGGGTATTTCCAAGAGTCATGC	gGCCTATAATATAATGACAGAGTAACA
Genotyping	rs10021205	ACGTTGGATGCTGCCCTTGAGCAAATGAAG	ACGTTGGATGGCTGTCCTCCATGTGTTTAG	CCTCCATGTGTTTAGCTAATCC

Genotyping	rs688099	ACGTTGGATGCTGAGAAGGTACCACTCAGC	ACGTTGGATGTCTGTCTGAAATGCCTACCC	ccCCTTGCTGGGTTTCTTTTTCG
Genotyping	rs16959059	ACGTTGGATGCAAAGCAAACCCAGCAGGTA	ACGTTGGATGGTCTCCTATGTAATCTCTTAG	AAAAGTTCAGAAAATTCAAAAATAAC
TaqMan		Primer	Probe	
Genotyping	rs2238126	F:CAGATGATTGATGTGGGCATATG	FAM-AACATACAGAAAGGTC (G allele)	
		R:GCAACATGTGTCATCTTGGGTTA	HEX-TAACATACAGAAAGATCTG (A allele)	
RT-qPCR	*ETV6*	F: AGGCCATCCGTGGATAATGTG	R: CGGTGATTTGTCGTGATAGGTGA	
RT-qPCR	*ACTINB*	F: CATGTACGTTGCTATCCAGGC	R: CTCCTTAATGTCACGCACGAT	
RT-qPCR	*18sRNA*	F: CAGCCACCCGAGATTGAGCA	R: TAGTAGCGACGGGCGGTGTG	
RT-qPCR	*HRPT1*	F: CCTGGCGTCGTGATTAGTGAT	R: AGACGTTCAGTCCTGTCCATAA	
RT-qPCR	*UBC*	F: CTGGAAGATGGTCGTACCCTG	R: GGTCTTGCCAGTGAGTGTCT	
RT-qPCR	*GAPDH*	F: GCACCGTCAAGGCTGAGAAC	R: TGGTGAAGACGCCAGTGGA	
EMSA	rs2238126 A	F: CATACAGAAAGATCTGGTTCTGA	R: TCAGAACCAGATCTTTCTGTATG	
	rs2238126 G	F: CATACAGAAAGGTCTGGTTCTGA	R: TCAGAACCAGACCTTTCTGTATG	
RT-qPCR	ChIP	F: ATGATTGATGTGGGCATA	R: AATAAATCACTGGGGAAG	
Knockdown	shRNA1	CCGGCCATAAGAACAGAACAAACATCTCGAGATGTTTGTTCTGTTCTTATGGTTTTTG		
	shRNA2	CCGGGCGCCACTACTACAAACTAAACTCGAGTTTAGTTTGTAGTAGTGGCGCTTTTTG		
	shRNA3	CCGGAGGAGCTGGATGAACAAATATCTCGAGATATTTGTTCATCCAGCTCCTTTTTTG		

Supplementary Table 11. Clinical characteristics of colorectal cancer tissues

No.	Age (years)	Sex	Site	Grade	Dukes stage
1	78	Male	Rectum	Intermediate	B
2	65	Male	Rectum	High	C
3	84	Male	Colon	Intermediate	B
4	57	Female	Rectum	Intermediate	B
5	42	Male	Colon	Intermediate	C
6	56	Female	Rectum	Intermediate	C
7	29	Female	Rectum	Low	B
8	55	Male	Rectum	Intermediate	C
9	49	Female	Rectum	Intermediate	A
10	38	Male	Colon	Intermediate	C
11	68	Male	Rectum	Intermediate	C
12	58	Female	Colon	High	B
13	66	Female	Colon	Intermediate	C
14	55	Female	Rectum	Intermediate	B
15	75	Male	Rectum	Intermediate	B
16	49	Male	Colon	Intermediate	B
17	79	Female	Rectum	Intermediate	B
18	79	Female	Colon	Intermediate	B
19	69	Female	Colon	Intermediate	C
20	63	Male	Colon	High	C
21	76	Male	Colon	Intermediate	B
22	68	Male	Rectum	Intermediate	B
23	52	Male	Colon	Intermediate	B
24	79	Female	Colon	Intermediate	B
25	47	Male	Colon	Intermediate	B
26	66	Female	Rectum	Intermediate	C
27	66	Male	Rectum	Intermediate	B
28	55	Male	Rectum	Low	B
29	63	Female	Rectum	Intermediate	C
30	74	Male	Colon	Intermediate	B
31	78	Male	Rectum	Intermediate	C
32	77	Female	Colon	Intermediate	B
33	63	Male	Colon	Intermediate	D
34	63	Male	Rectum	Intermediate	C
35	56	Male	Colon	Low	C
36	57	Female	Colon	Intermediate	C
37	49	Female	Rectum	Intermediate	B
38	68	Male	Rectum	Intermediate	C
39	78	Female	Colon	Intermediate	C
40	61	Female	Rectum	Intermediate	B
41	54	Female	Rectum	Intermediate	C
42	56	Female	Colon	Intermediate	B

43	74	Female	Rectum	Intermediate	B
44	68	Male	Rectum	Intermediate	B
45	84	Male	Colon	Intermediate	A
46	76	Male	Rectum	Intermediate	B
47	54	Female	Rectum	Intermediate	A
48	69	Female	Colon	Intermediate	B
49	83	Male	Colon	Intermediate	B
50	56	Male	Rectum	Intermediate	B
51	39	Female	Rectum	Intermediate	C
52	58	Female	Rectum	Intermediate	B
53	59	Male	Rectum	Low	C
54	68	Male	Rectum	Intermediate	B
55	57	Male	Colon	Intermediate	B
56	61	Female	Rectum	Intermediate	C
57	64	Male	Colon	Intermediate	B
58	56	Female	Rectum	Intermediate	C
59	44	Female	Rectum	Intermediate	C
60	80	Male	Rectum	Intermediate	C
61	49	Male	Colon	High	C
62	74	Female	Rectum	Intermediate	B
63	56	Female	Colon	Intermediate	C
64	40	Female	Colon	High	A
65	29	Male	Rectum	Low	C
66	54	Male	Rectum	Intermediate	A
67	76	Male	Colon	Intermediate	B
68	59	Male	Colon	Intermediate	C
69	88	Male	Colon	Intermediate	C
70	78	Female	Colon	Intermediate	B
71	42	Female	Rectum	High	B
72	54	Female	Rectum	Intermediate	B
73	43	Male	Rectum	Intermediate	B
74	53	Female	Rectum	Intermediate	A
75	65	Male	Colon	Intermediate	C
76	56	Female	Colon	Intermediate	B
77	56	Male	Rectum	Intermediate	B
78	69	Male	Colon	Intermediate	B
79	62	Female	Rectum	Intermediate	B
80	67	Female	Colon	High	B
81	73	Female	Colon	Intermediate	C
82	79	Male	Colon	Intermediate	B
83	73	Male	Rectum	Intermediate	B
84	68	Male	Rectum	Intermediate	B
85	69	Male	Colon	Low	C
86	82	Male	Rectum	Intermediate	B

87	75	Male	Colon	Intermediate	B
88	82	Male	Colon	High	B
89	57	Male	Colon	Intermediate	B
90	53	Female	Colon	Intermediate	B
91	53	Female	Colon	Intermediate	B
92	54	Male	Rectum	Intermediate	C
93	59	Male	Colon	Intermediate	B
94	59	Male	Colon	Intermediate	C
95	56	Female	Rectum	Intermediate	C
96	58	Male	Colon	Intermediate	B
97	60	Male	Colon	Intermediate	C
98	55	Female	Rectum	Intermediate	D
99	78	Male	Rectum	Intermediate	D
100	67	Female	Colon	Intermediate	B
101	42	Female	Colon	Intermediate	C
102	54	Male	Rectum	High	B
103	58	Female	Colon	High	C
104	81	Male	Colon	Intermediate	B
105	39	Male	Rectum	Intermediate	B
106	64	Male	Rectum	Intermediate	B
107	75	Female	Colon	Intermediate	B
108	73	Male	Rectum	Intermediate	B
109	48	Female	Rectum	Intermediate	C
110	49	Male	Colon	Low	B
111	54	Male	Rectum	Intermediate	A
112	46	Male	Rectum	Intermediate	C
